# SOX9 is a novel cancer stem cell marker surrogated by osteopontin in human hepatocellular carcinoma

**DOI:** 10.1038/srep30489

**Published:** 2016-07-26

**Authors:** Takayuki Kawai, Kentaro Yasuchika, Takamichi Ishii, Yuya Miyauchi, Hidenobu Kojima, Ryoya Yamaoka, Hokahiro Katayama, Elena Yukie Yoshitoshi, Satoshi Ogiso, Sadahiko Kita, Katsutaro Yasuda, Ken Fukumitsu, Junji Komori, Etsuro Hatano, Yoshiya Kawaguchi, Shinji Uemoto

**Affiliations:** 1 Department of Surgery, Graduate School of Medicine, Kyoto University, Kyoto, Japan; 2 Center for iPS Cell Research and Application (CiRA), Kyoto University, Kyoto, Japan

## Abstract

The current lack of cancer stem cell (CSC) markers that are easily evaluated by blood samples prevents the establishment of new therapeutic strategies in hepatocellular carcinoma (HCC). Herein, we examined whether sex determining region Y-box 9 (SOX9) represents a new CSC marker, and whether osteopontin (OPN) can be used as a surrogate marker of SOX9 in HCC. In HCC cell lines transfected with a SOX9 promoter-driven enhanced green fluorescence protein gene, FACS-isolated SOX9^+^ cells were capable of self-renewal and differentiation into SOX9^−^ cells, and displayed high proliferation capacity *in vitro*. Xenotransplantation experiments revealed that SOX9^+^ cells reproduced, differentiated into SOX9^−^ cells, and generated tumors at a high frequency *in vivo*. Moreover, SOX9^+^ cells were found to be involved in epithelial-mesenchymal transition (EMT) and activation of TGFb/Smad signaling. Gain/loss of function experiments showed that SOX9 regulates Wnt/beta-catenin signaling, including cyclin D1 and OPN. Immunohistochemistry of 166 HCC surgical specimens and serum OPN measurements showed that compared to SOX9^−^ patients, SOX9^+^ patients had significantly poorer recurrence-free survival, stronger venous invasion, and higher serum OPN levels. In conclusion, SOX9 is a novel HCC-CSC marker regulating the Wnt/beta-catenin pathway and its downstream target, OPN. OPN is a useful surrogate marker of SOX9 in HCC.

The similarity between the behavior of malignant cells and that of embryonic cells has resulted in the notion of “cancer stem cells (CSCs)”. Similar to embryonic stem/precursor cells in organogenesis, it has been reported that in various malignancies^1^,^2^,^3^,^4^,^5^,^6^, CSCs possess the ability to self-renew and differentiate into heterogeneous progenies, with high motility and proliferation rates^7^,^8^. In addition, the properties of CSCs include an ability to initiate tumor formations by xenotransplantation and resistance to chemotherapy and radiotherapy^9^,^10^. Therefore, identification of CSC markers and efforts to elucidate their relevance in cell signaling pathways potentially lead to the establishment of new therapeutic strategies. In hepatocellular carcinoma (HCC), various molecules expressed during hepatic organogenesis such as epithelial cell adhesion molecule (EpCAM), cluster of differentiation (CD) 90, CD133, CD24, CD13, sal-like protein 4 (SALL4), and keratin 19 (K19), have been reported as CSC markers, and the expressions of these markers have been shown to correlate with a poor prognosis of the patients^11^,^12^,^13^,^14^,^15^,^16^,^17^. However, there is still a lack of an easily identifiable CSC marker that could be evaluated by blood samples, and this has prevented us from fully predicting the patients’ outcomes or evaluating the therapeutic efficacy in patients with HCC.

Sex determining region Y-box 9 (SOX9) is involved in the organogenesis of several tissues and organs, including the testis, heart, lung, pancreas, hair follicles, retina, and the central nervous system^18^,^19^,^20^,^21^,^22^,^23^,^24^. In general, its function is to maintain cells at an undifferentiated state during development, and connected with many cell signaling pathways such as the Notch, transforming growth factor beta (TGFb)/Smad, and Wnt/beta-catenin pathways^25^,^26^,^27^. In hepatogenesis, SOX9 expression is first detected in the specification of cholangiocytes; bi-potent SOX9^−^ negative hepatoblasts lining the portal vein asymmetrically divide and differentiate to form SOX9^+^ cholangiocytes on the portal side and SOX9^−^ negative hepatoblasts on the parenchymal side^23^. After the maturation step, in which TGFb/Smad signaling is activated, the biliary tube becomes entirely composed of SOX9^+^ cholangiocytes^26^. Thus, SOX9 expression is confined to the bile duct, while hepatocytes do not express SOX9 during embryogenesis; this expression pattern persists in adulthood^28^. Notably, previously reported HCC-CSCs markers such as EpCAM, CD90, CD133, CD24, CD13, SALL4, and K19 are all expressed in the embryonic liver, while their expressions are not normally detected in adult hepatocytes. Considering the results of two independent lineage tracing studies, which demonstrated that embryonic SOX9^+^ cells possess an ability to differentiate into hepatocytes^29^,^30^, we hypothesized that SOX9 is an excellent candidate CSC marker in human HCC. To test this hypothesis, we sorted SOX9^+^ cell population from several human HCC cell lines and analyzed their CSC characteristics. While our analyses were in progress, Liu *et al*. reported that SOX9 is highly expressed in Nanog^+^ HCC cells and SOX9 regulates self-renewal/tumorigenicity in HCC^31^. However, it is still unknown what signals are responsible for the maintenance of stem cell properties in SOX9^+^ HCC-CSCs. In this study, we demonstrate that SOX9 is required for the activation of TGFb/Smad pathway and that Wnt/beta-catenin signaling is constitutively and more highly activated in SOX9^+^ cells compared to SOX9^−^ population. Moreover, we provide evidence that osteopontin (OPN), another downstream target of the Wnt/beta-catenin pathway^32^, is regulated by SOX9 and highly expressed in the blood samples from the patients bearing SOX9^+^ HCC, putting OPN as a surrogate marker of SOX9 in human HCCs.

## Results

### EGFP-marking of the SOX9^+^ cell populations in heterogeneous HCC cell lines

Our RT-PCR analyses showed that all HCC lines tested (Huh7, HLF, PLC/PRF/5, and Hep3B) expressed SOX9 (Supplemental Fig. 1A). Histologically, all cell lines contained both SOX9-expressing and non-expressing cells. To mark SOX9^+^ and SOX9^−^ cells, we transfected the SOX9-EGFP reporter vector into the four cell lines (Supplemental Fig. 1B). Double staining of SOX9 and GFP confirmed that our selective GFP-labeling successfully marked SOX9-expressing cells, with an efficiency of >95% (Supplemental Fig. 1C). The ratios of SOX9^+^ cells were different among all cell lines, and this was reflected by our FACS results; 16.9% ± 3.5% of the Huh7 cells, 30.8% ± 2.9% of the HLF cells, 23.5% ± 4.8% of the PLC/PRF/5 cells, and 14.2% ± 3.7% of the Hep3B cells (*n* = 3) expressed EGFP (Supplemental Fig. 1D). PCR analyses confirmed an equal copy number of the reporter gene detected in the sorted EGFP^−^ and EGFP^+^ cells (Supplemental Fig. 1E). Expressions of previously reported HCC-CSCs markers were evaluated in SOX9^+^/SOX9^−^ cells (Supplemental Fig. 1F).

### CSC-like properties of SOX9^+^ cells *in vitro*

**Figure 1:**
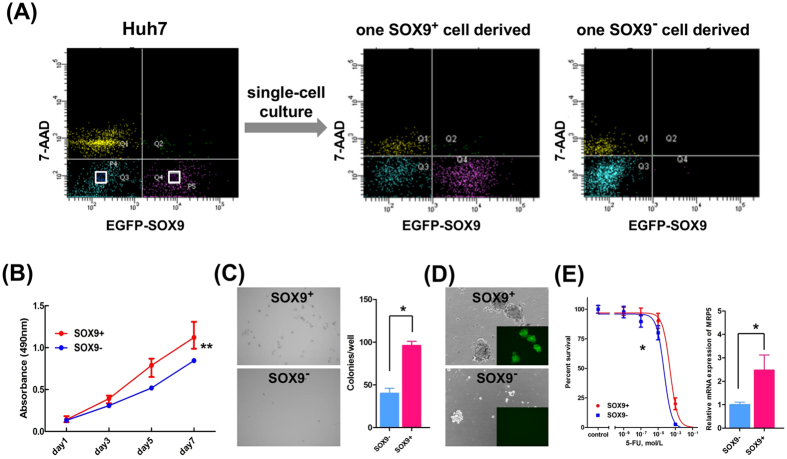
Cancer stem cell properties of SOX9^+^ Huh7 cells *in vitro*. (**A**) Single-cell culture of SOX9^+^ and SOX9^−^ Huh7 cells. FACS analyses revealed that isolated SOX9-EGFP^+^ cell differentiated both EGFP^+^ and EGFP^−^ cell fractions (middle panel), whereas isolated SOX9-EGFP^−^ cell only to EGFP^−^ cell fraction (right panel). (**B**) Cell proliferation assays showed SOX9^+^ cells proliferate more than SOX9^−^ cells (repeated-measures ANOVA, **P < 0.01). (**C**) Microscopic appearance and the colony numbers in the anchorage-independent growth assay (Student’s *t*-test, *P < 0.05). (**D**) Phase-contrast images in the sphere-forming assay. (**E**) IC_50_ of 5-FU in SOX9^+^ and SOX9^−^ cells (left panel, *F*-test, *P < 0.05) and qRT-PCR analyses of MRP5 in SOX9^+^ and SOX9^−^ cells (right panel, Student’s *t*-test, *P < 0.05).

To test if the SOX9^+^ cell population behaved as CSCs, we first performed single-cell culture analyses to examine its self-renewal activity and multi- or bi-potency. Notably, the single SOX9^+^ Huh7 cell reproducibly differentiated to SOX9^+^ and SOX9^−^ cells, whereas the single SOX9^−^ Huh7 cell generated only a SOX9^−^ cell fraction (Fig. 1A). Next, we compared the SOX9^+^ and SOX9^−^ Huh7 cells in terms of their proliferation, anchorage-independent growth, and sphere-forming ability. We found that SOX9^+^ cells showed higher proliferation activity (Fig. 1B) and formed more colonies in soft agar (Fig. 1C). In addition, sorted SOX9^+^ Huh7 cells showed superior sphere-forming ability; large spheres formed in higher frequency compared to in SOX9^−^ Huh7 cells (Fig. 1D).

CSCs are known to show strong resistance to chemotherapy^9^. Herein, we found that SOX9^+^ Huh7 cells were significantly more resistant to 5-fluorouracil (5-FU) than SOX9^−^ Huh7 cells, with half-maximal inhibitory concentrations (IC_50_) values of 2.5 × 10^−4^ M and 4.9 × 10^−5^ M for SOX9^+^ and SOX9^−^ Huh7 cells, respectively (Fig. 1E). To explain the observed 5-FU resistance, our quantitative reverse transcription (qRT)-PCR analysis revealed that the expression of multidrug-resistance protein-5 (MRP5), a key drug transporter of 5-FU^33^, was significantly higher in SOX9^+^ cells (Fig. 1E).

Based on these findings, we concluded that SOX9^+^ Huh7 cells have the ability to self-duplicate and differentiate into other cell type, thus showing higher malignant potential than SOX9^−^ Huh7 cells. These results were consistent in all HCC cell lines tested, including the HLF, PLC/PRF/5, and Hep3B cell lines (Supplemental Fig. 2B–E). Taken together, these findings indicate that SOX9^+^ HCC cells possess the characteristics of CSCs *in vitro*, including the ability of bi-potent differentiation with self-renewal, high proliferation, colony and sphere formation, and resistance to 5-FU.

### Xenotransplantation into immunodeficient mice

**Figure 2:**
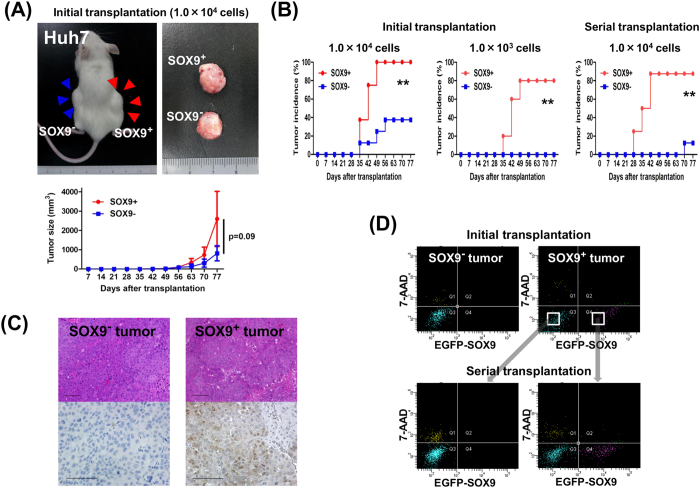
Cancer stem cell properties of SOX9^+^ Huh7 cells *in vivo*. (**A**) Xenotransplantation of 1.0 × 10^4^ SOX9^+^ and SOX9^−^ cells in NOD/SCID mice, the formed tumor, and the tumor development of transplanted SOX9^+^ or SOX9^−^ cells (repeated-measures ANOVA, *P* = 0.09). Data are shown as the mean ± SD. (**B**) Tumor incidence in initial and serial transplantation. Note the higher incidence of tumor formation by SOX9^+^ cell transplantation (log-rank test, **P < 0.01). (**C**) Immunohistochemical analysis of tumor. Note that SOX9^+^ and SOX9^−^ cells detected in SOX9^+^ cell-driven tumor. Scale bar represents 100 μm. (**D**) FACS analyses of dissociated tumor cells confirmed bi-potent differentiation ability conserved in serial transplantation of SOX9^+^ cells.

To explore the CSC properties of SOX9^+^ cells *in vivo*, we transplanted SOX9^+^ or SOX9^−^ cells, derived from a single culture of SOX9^+^ Huh7 cells, into the backs of NOD/SCID mice. When 1.0 × 10^4^ cells were transplanted, SOX9^+^ cells reproducibly formed larger tumors than SOX9^−^ cells although the difference was not statistically significant (Fig. 2A). Notably, the tumor initiating frequency was significantly higher in SOX9^+^ cells compared to SOX9^−^ cells as calculated by the limiting dilution experiments (Supplemental Tables 1 and 2). The lower tumor-forming ability of SOX9^−^ cells was confirmed in the serial transplantation of 1.0 × 10^4^ cells (87.5% in SOX9^+^ cells and 12.5% in SOX9^−^ cells) (Fig. 2B, Supplemental Table 1). Consistent with the *in vitro* single cell culture experiments, immunohistological and FACS analyses revealed that the tumors originating from SOX9^+^ cells consisted of both SOX9^+^ and SOX9^−^ cell fractions, whereas those from SOX9^−^ cells contained only a SOX9^−^ cell fraction (Fig. 2C,D). This bi-potent differentiation ability of SOX9^+^ tumor was confirmed by FACS analyses of the tissue obtained from the serial transplantation experiments (Fig. 2D). We obtained similar results with the HLF, PLC/PRF/5, and Hep3B cell lines (Supplemental Fig. 3A–D, Supplemental Tables 1 and 2). These results clearly demonstrate that SOX9^+^ cells possess the capacity to replicate, to generate heterogeneous lineages of cancer cells, and to initiate tumor formations *in vivo*.

### Involvement of SOX9 expression in EMT through TGFb/Smad signaling

**Figure 3:**
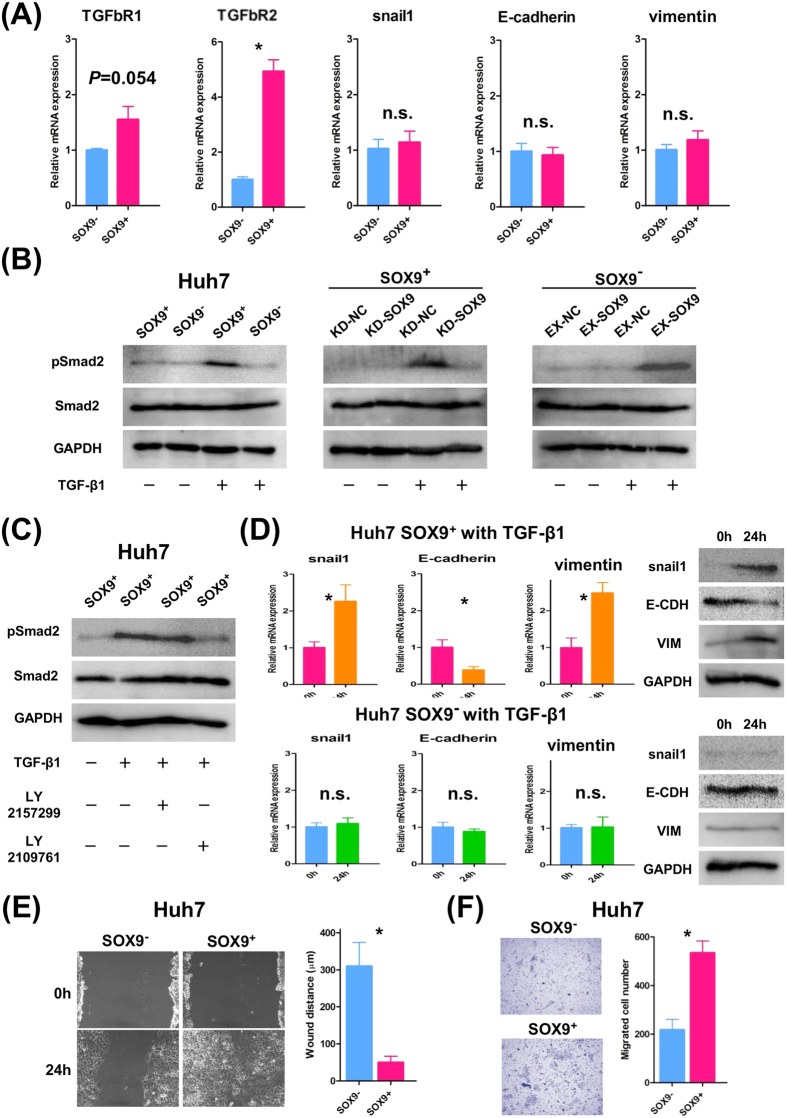
TGFb-induced EMT phenotype in SOX9^+^ Huh7 cells. (**A**) qRT-PCR analysis of TGFb receptors and EMT-related genes in SOX9^+^ and SOX9^−^ cells without TGFb stimulation (Student’s *t*-test, *P < 0.05). Data are shown as the mean ± SD. (**B**) Western blot analysis showed TGFb-induced pSmad2 upregulation in SOX9^+^ but not in SOX9^−^ cells (left panel), and regulation of TGFb-induced pSmad2 upregulation by SOX9 (middle and right panel). (**C**) Western blot analysis showed TGFb-induced pSmad2 upregulation in SOX9^+^ cells was suppressed by LY2109761 but not LY2157299. (**D**) qRT-PCR and western blot analyses showed elevation of snail1 and vimentin accompanied by E-cadherin downregulation, caused by TGFb stimulation in SOX9^+^ cells (Student’s *t*-test, SOX9^+^ Huh7 cells; *P < 0.05, SOX9^−^ Huh7 cells; not significant). Data are shown as the mean ± SD. (**E**) Wound-healing assay of SOX9^+^ and SOX9^−^ cells with TGFb stimulation (Student’s *t*-test, *P < 0.05). (**F**) Migration assay of SOX9^+^ and SOX9^−^ cells with TGFb stimulation (Student’s *t*-test, *P < 0.05).

Recently, the pathogenesis of EMT has been reported to be closely connected to the CSC properties^34^. It is well known that TGFb/Smad signaling plays a central role in EMT; activation of this signaling pathway upregulates snail expression, which in turn triggers EMT, resulting down-regulation of E-cadherin and increased expression of vimentin^35^,^36^. Our qRT-PCR assays revealed that SOX9^+^ Huh7 cells had significantly higher TGFbR2 expression than SOX9^−^ cells at the steady state, whereas there were no significant differences in the expression of snail1, E-cadherin, or vimentin without TGFb stimulation (Fig. 3A). Upon TGFb stimulation, SOX9^+^ cells induced the higher pSmad2 expression in than in SOX9^−^ cells (Fig. 3B). Since TGFb-stimulated pSmad2 induction was suppressed by SOX9 knockdown in SOX9^+^ cells and rescued by transgene-based SOX9 overexpression in SOX9^−^ cells, activation of TGFb/Smad signaling is SOX9-dependent (Fig. 3B). In accordance with the higher expression of TGFbR2 at the steady state, activation of TGFb/Smad signaling is mediated via Type 2 receptor, since dual TGFbR1/R2 inhibitor LY2109761 but not Type 1 receptor-specific inhibitor LY2157299 suppressed pSmad2 induction in SOX9^+^ cells (Fig. 3C). Remarkably, upon TGFb stimulation (10 ng/ml for 24 h), SOX9^+^ Huh7 cells, but not SOX9^−^ cells, developed an EMT expression profile including down-regulation of E-cadherin and up-regulation of snail1 and vimentin expressions (Fig. 3D). Additionally, *in vitro* wound healing and migration assays revealed that upon TGFb stimulation, SOX9^+^ cells displayed significantly greater motility than SOX9^−^ cells (Fig. 3E,F). These results indicate that TGFb/Smad signaling is activated more easily and efficiently in SOX9^+^ Huh7cells than in SOX9^−^ population. Significance of TGFb/Smad signaling in CSC properties is supported by our functional rescue experiments that, upon the stimulation of the higher dose of TGFb1 (30 ng/ml), sphere forming and proliferation ability of SOX9^−^ Huh7 cells was accelerated to the same level of non-treated SOX9^+^ cells (compare Supplemental Fig. 4 and Fig. 1B,D). Similar results were obtained with the HLF, PLC/PRF/5, and Hep3B cell lines (data not shown).

### Activation of the Wnt/beta-catenin pathway in SOX9-expressing cells

**Figure 4:**
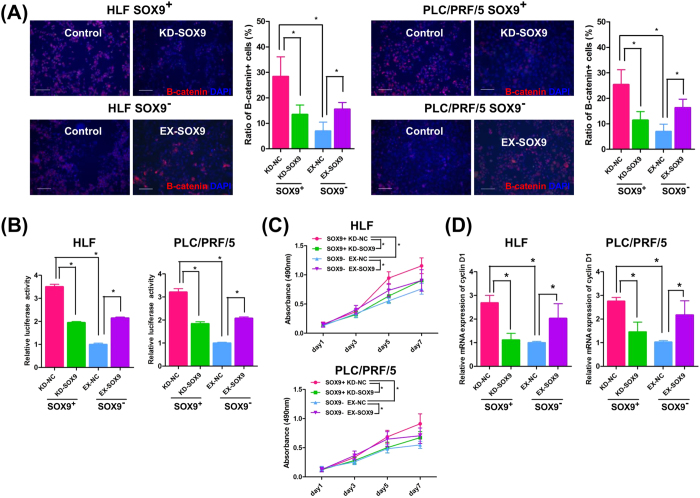
SOX9 activates the Wnt/beta-catenin pathway in HLF and PLC/PRF/5 cells. (**A**) Beta-catenin staining. Note the nuclear localized, activated beta-catenin expression in the steady state SOX9^+^ cells (Control) and its reduction by SOX9 knockdown (KD-SOX9). Exogenously introduced SOX9 (EX-SOX9) accelerated beta-catenin activation in SOX9^−^ cells (Student’s *t*-test, *P < 0.05). Scale bar represents 100 μm. (**B**) TCF/LEF luciferase assay with SOX9-gain/loss of function experiments (Student’s *t*-test, *P < 0.05). (**C**) Cell proliferation assays with SOX9-gain/loss of function experiments (repeated-measures ANOVA, *P < 0.05). (**D**) qRT-PCR analysis of cyclin D1 with SOX9-gain/loss of function experiments (Student’s *t*-test, *P < 0.05). Data are shown as the mean ± SD. KD-NC; control knockdown, KD-SOX9; SOX9 knockdown, EX-NC; control overexpression, EX-SOX9; SOX9 overexpression.

Another signaling pathway involved in CSC maintenance is the Wnt/beta-catenin pathway^34^,^37^. To test the activity of the Wnt/beta-catenin pathway in SOX9^+^/SOX9^−^ cells, we selected the HLF and PLC/PRF/5 cell lines, owing to their relatively high SOX9 expression among the four cell lines (Supplemental Fig. 1A). Compared to the SOX9^−^ population, we found a more nuclear localization of beta-catenin in SOX9^+^ HLF and PLC/PRF/5 cells, indicating activation of the Wnt/beta-catenin pathway in these cells at the steady state (indicated as control in Fig. 4A). To confirm that SOX9 regulates the Wnt/beta-catenin pathway, we performed gain/loss of SOX9 function experiments. In SOX9^+^ HLF and PLC/PRF/5 cells, siRNA-based SOX9 knockdown significantly decreased the ratio of activated beta-catenin^+^ cells, the TCF/LEF activity, the proliferation ability, and the cyclin D1 expression (Fig. 4A−D). On the other hand, SOX9 overexpression in SOX9^−^ HLF and PLC/PRF/5 cells consistently and significantly increased the ratio of activated beta-catenin^+^ cells, the TCF/LEF activity, the proliferation ability, and the cyclin D1 expression (Fig. 4A–D). Involvement of Wnt/beta-catenin pathway in CSC property is supported by the observation that SOX9^−^ cells showed accelerated sphere forming and proliferation activity by the stimulation of CHIR99021, an activator of Wnt/beta-catenin pathway, to the same level of non-treated SOX9^+^ cells (compare Supplemental Fig. 5 and Fig. 1B,D).

**Figure 5:**
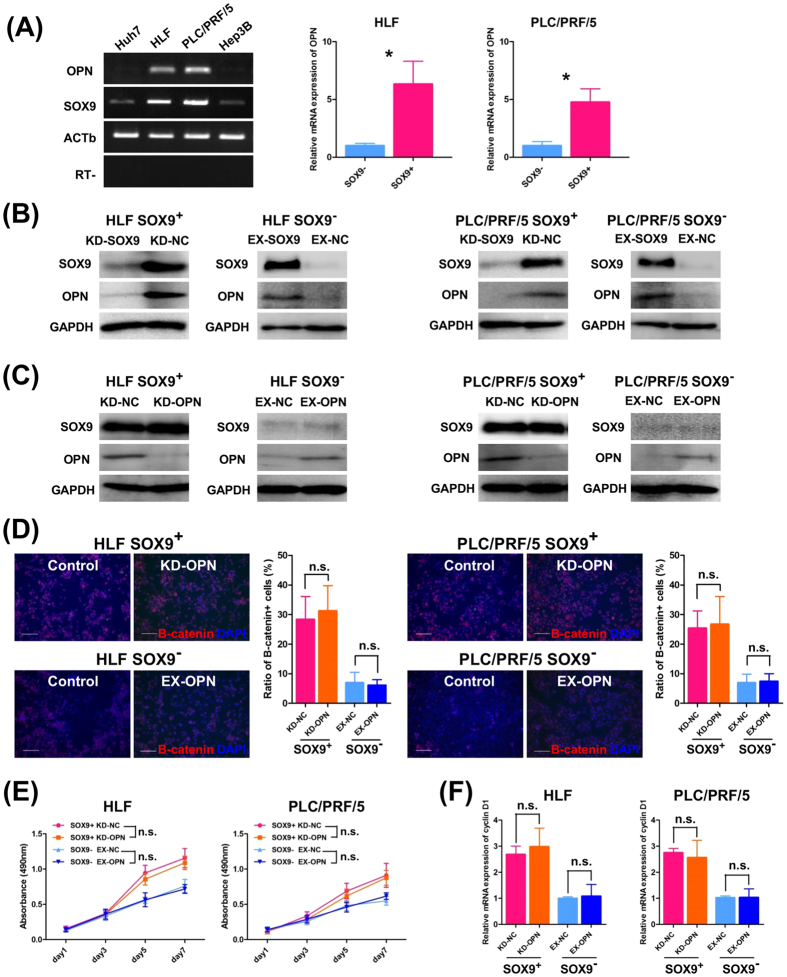
SOX9 regulates OPN expression in HLF and PLC/PRF/5 cells. (**A**) RT-PCR analysis of SOX9/OPN in HCC cell lines (left) and qRT-PCR analysis of OPN in SOX9^+^/SOX9^−^ HLF and PLC/PRF/5 cells (right, Student’s *t*-test, *P < 0.05). Data are shown as the mean ± SD. (**B**) Western blot analysis of SOX9 and OPN in SOX9^+^/SOX9^−^ cells with SOX9-gain/loss of function experiments revealed that SOX9 induced OPN expression. (**C**) Western blotting with OPN-gain/loss of function experiments showed that OPN did not regulate SOX9 expression. (**D–F**) OPN did not affect the Wnt/beta-catenin activity including nuclear-localization of beta-catenin, cell proliferation, and the expression of cyclin D1 (**D,F** Student’s *t*-test, not significant, **E**; repeated-measures ANOVA, not significant). Scale bar represents 100 μm.

A previous report suggested OPN, a component of the extracellular matrix, as a downstream target of the Wnt/beta-catenin pathway^32^ and its expression has been demonstrated to correlate with SOX9 in liver fibrosis^38^,^39^. These findings prompted us to examine whether there exists mutual regulation between SOX9 and OPN that in turn activates the Wnt/beta-catenin pathway. In the present study, the level of OPN expression was found to correlate with the SOX9 expression in all four HCC cell lines tested, and qRT-PCR assays revealed that sorted EGFP^+^ cells, that is, SOX9^+^ cells, showed significantly higher OPN expression than EGFP^−^/SOX9^−^ cells in the HLF and PLC/PRF/5 cell lines (Fig. 5A). Furthermore, we found that OPN expression was suppressed by SOX9 knockdown and elevated by SOX9 overexpression (Fig. 5B). In contrast, gain/loss of OPN function did not affect SOX9 expression (Fig. 5C) and caused no apparent changes in the ratio of activated beta-catenin^+^ cells, the proliferation ability, or the cyclin D1 expression (Fig. 5D–F). Based on these results, we concluded that SOX9, not OPN, activates the Wnt/beta catenin pathway in HCC cells and that mutual regulation between SOX9 and OPN does not exist.

### SOX9/OPN expressions in human HCC surgical specimens and their clinicopathological significance

**Figure 6:**
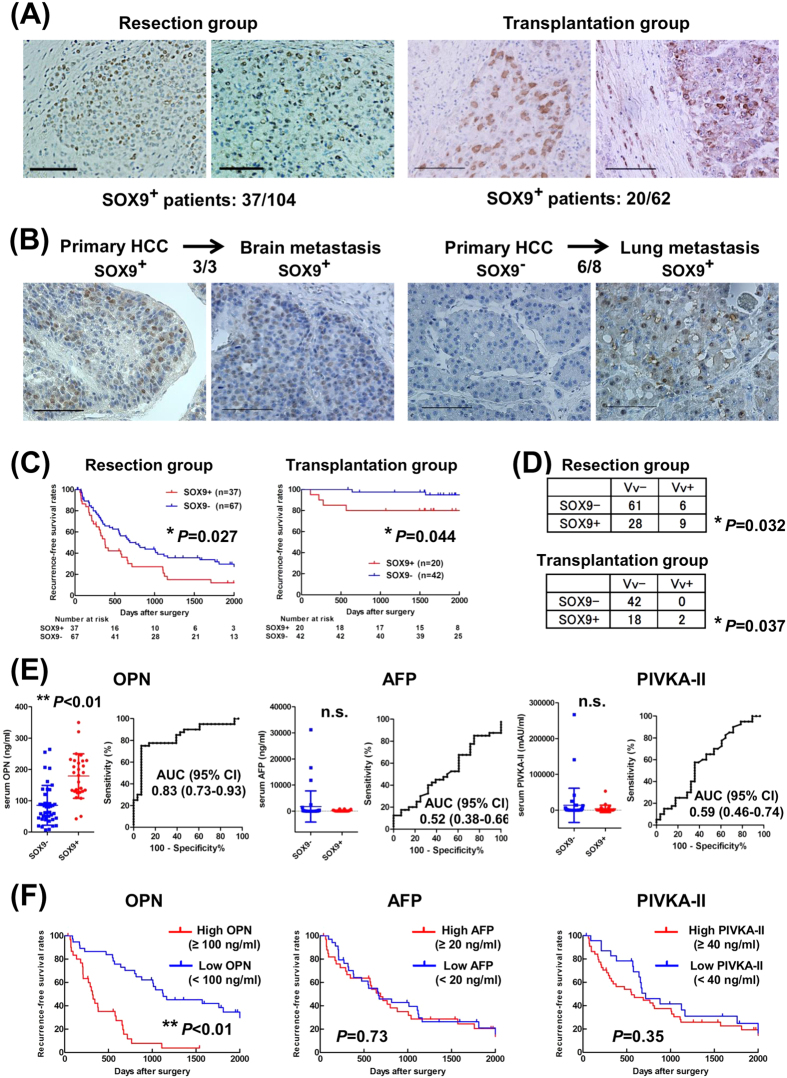
SOX9 and OPN expression in human HCC. (**A**) Representative pictures of SOX9-expressing tumor in the resection group (left) and two patients in the transplantation group (right). (**B**) High incidence of SOX9 expression from SOX9^+^ (left) and SOX9^−^ HCCs (right). (**C**) Recurrence-free survival rates. Note the significantly higher recurrence (log-rank test, *P = 0.027 in the resection group (left), *P = 0.044 in the transplantation group (right)). (**D**) Correlation between SOX9 expression and venous invasion (Fisher’s exact test, resection group; *P = 0.032, transplantation group; *P = 0.037). (**E**) Serum levels and ROC curve evaluating the performance of serum OPN, AFP, and PIVKA-II levels in predicting SOX9 expression in HCC. Each line in left panel indicates median levels with 95% confidence interval (CI). SOX9^+^ patients had significantly higher serum OPN than SOX9^−^ patients (Mann-Whitney *U* test, **P < 0.01). (**F**) Recurrence-free survival rates of 68 HCC patients in terms of serum level of OPN or AFP or PIVKA-II (log-rank test, OPN; **P < 0.01, AFP; *P* = 0.73, PIVKA-II; *P* = 0.58). Scale bar represents 100 μm (**A,B**).

To examine the SOX9 expression in human HCC clinical samples, 166 surgically resected primary HCC tumors, including 104 cases of hepatectomy and 62 cases of liver transplantation, and 11 metastatic HCC tumors, were subjected to immunohistochemistry. SOX9 expression in primary HCC nodules was observed in 37/104 (36%) cases in the resection group and in 20/62 (32%) cases in the transplantation group (Fig. 6A). Notably, we observed a higher incidence of SOX9 expression (9/11 cases [82%]) in the metastatic nodules. In detail, 3/3 metastatic nodules originating from SOX9^+^ primary lesions expressed SOX9; among the 8 cases in which apparent SOX9 immunoreactivity was not detected in the primary lesion, 6 metastatic nodules showed SOX9 expression (Fig. 6B and Supplemental Table 3), suggesting that SOX9 expression is associated with postoperative tumor recurrence/metastasis in HCC.

Supporting this notion, the SOX9^+^ patients had significantly lower relapse-free survival (RFS) with the median RFS being 378 days for SOX9^+^ patients and 765 days for SOX9^−^ patients in the resection group, and 2344 days and 2746 days in SOX9^+^ and SOX9^−^ patients, respectively, in the transplantation group (Fig. 6C). However, the overall survival did not show significant differences according to the SOX9 expression status (Supplemental Tables 4 and 5). The significance of SOX9 expression in predicting postoperative recurrence was confirmed by the log-rank test and multivariate analysis in the resection and transplantation groups (Table 1 and Supplemental Tables 4 and 5). Interestingly, SOX9 expression was found to correlate with venous invasion in both the resection and transplantation groups (Fig. 6D, resection group; *P* = 0.032, transplantation group; *P* = 0.037).

Our finding that SOX9 regulates OPN expression in HCC cell lines (Fig. 5A–C) prompted to test if OPN could be used as a surrogate marker of SOX9 in human HCCs. As shown in Supplemental Fig. 4, we observed a significant correlation between the expressions of SOX9 and OPN in the human HCC samples (*P* < 0.01). Especially, in metastatic HCC nodules, OPN expression was detected in 9/9 (100%) and 0/2 (0%) patients with SOX9^+^ and SOX9^−^ HCC primary nodules, respectively. In addition, we measured the serum OPN level using the available 68 blood samples in the resection group and found that it was significantly higher in SOX9^+^ (n = 28, mean = 179 ng/mL) than in SOX9^−^ patients (n = 40, mean = 86 ng/mL) (Fig. 6E). It should be noted that receiver operating characteristics (ROC) analysis revealed that the serum OPN level was a more sensitive indicator of SOX9 expression in the tumors compared to alpha-fetoprotein (AFP) and protein induced by vitamin K antagonists-II (PIVKA-II) (Fig. 6E and Supplemental Table 6). Consistent with the finding that SOX9 expression in the tumor was a good indicator of tumor recurrence, the serum OPN, but not AFP or PIVKA-II, level showed a significant correlation with the RFS in our analyses (Fig. 6F).

## Discussion

In this study, we used SOX9-promoter driven EGFP-marked cells to isolate SOX9^+^ populations in human HCC cell lines and demonstrated that SOX9^+^ cells possess the ability to self-renew and to differentiate into SOX9^−^ cells. Moreover, SOX9^+^ cells showed a higher proliferation ability, colony/sphere forming ability, and resistance to 5-FU *in vitro*. In addition, by xenotransplantation into NOD/SCID mice, we revealed that SOX9^+^ cells formed larger tumors at a higher frequency, retaining the differentiation ability to SOX9^+^ and SOX9^−^ populations. Taken together, these results clearly show that SOX9 fulfills various aspects of CSCs in human HCC cell lines.

Considering the pivotal functions of various signaling pathways in the maintenance and differentiation of embryonic stem/progenitor cells, including the Notch, TGFb/Smad signaling, and Wnt/beta-catenin signaling pathway^21^,^23^,^25^,^26^,^27^, it can be easily imagined that such signals also function in CSCs. As a matter of fact, the activation of TGFb/Smad signaling causes EMT both in the embryonic formation of several tissues/organs and during cancer progression including HCC^36^,^37^,^40^. Herein, we demonstrated that SOX9^+^ cells shed their epithelial properties and acquire more migratory mesenchymal cell-like characteristics by TGFb stimulation, indicating that SOX9^+^ cells possess high metastatic potential.

Previous reports have shown the involvement of the Wnt/beta-catenin pathway in controlling the proliferation and differentiation of embryonic stem/progenitor cells and CSCs^41^,^42^. In HCC-CSCs, it has been previously reported that Wnt/beta-catenin pathway, a major signaling pathway affecting hepatocarcinogenesis^43^, regulates EpCAM expression^11^. Reduction of EpCAM expression by siRNA suppressed tumor invasion, sphere formation, and the incidence of tumor formation by xenotransplantation in HCC cell lines^11^; however, it remains unclear whether EpCAM directly regulates the Wnt/beta-catenin pathway activity. As to the role of SOX9 in the Wnt/beta-catenin pathway, there seems distinct mechanism were reported; SOX9 negatively regulates Wnt/beta-catenin pathway in chondrocytes differentiation^44^, while another report showed that SOX9 is activated as a downstream target of Wnt/beta-catenin pathway in the initial step or basal cell carcinoma formation^45^. In the present study, we provided evidence that SOX9 activates Wnt/beta-catenin pathway in human HCC cell lines; our gain/loss of SOX9 function experiments showed increase/decrease in nuclear-localized beta-catenin expression and its downstream targets including cyclin D1 and OPN. These findings strongly suggest SOX9 as a regulator of HCC-CSC characteristics through Wnt/beta-catenin pathway.

Our analyses using human samples supported the relevance of SOX9 in the maintenance of CSC properties. Compared to those with SOX9^−^ tumors, patients with SOX9^+^ tumors exhibited significantly shorter RFS, with a higher incidence of venous invasion. Reflecting this, SOX9 expression was more frequently detected in metastatic HCC lesions of patients with both SOX9^+^ and SOX9^−^ primary HCCs; about 90% in the metastatic regions and 30% in the primary HCCs. The reason why SOX9 expression was detected in metastatic lesions from SOX9^−^ primary tumors may be the threshold of immunoreactivity of SOX9 staining; that is, there may have been a small number of SOX9^+^ HCC cells in the primary tumor that were not detected by immunohistochemistry. Alternatively, transdifferentiation into SOX9^+^ cells may occur in the process of metastasis in which EMT and MET play pivotal roles, and that could not recapitulated in the culture experiments.

Expression of OPN in the liver is connected with fibrosis and chronic inflammation and it has been reported to be a useful marker of early HCC and postoperative recurrence^46^,^47^. Herein, we showed significance of OPN in HCC-CSCs; we provided evidence that OPN is regulated by SOX9-mediated Wnt/beta-catenin pathway in HCC cell lines. Furthermore, we revealed that SOX9 expression was significantly correlated with the serum OPN level in human cases, and that its efficacy in identifying SOX9 expression in the tumors was superior to conventional tumor markers such as AFP and PIVKA-II.

In conclusion, the results of our study indicated that SOX9 is a novel HCC-CSC marker regulating the Wnt/beta-catenin pathway and its downstream target OPN, and that OPN is a useful surrogate marker of SOX9 in HCC. We believe that further studies of SOX9-related mechanisms will contribute to the development of novel therapeutic approaches in HCC treatment.

## Methods

### Patients

This study included 166 consecutive patients with HCC confirmed by pathologic analyses who had undergone hepatic resection between January 2005 and December 2006 (resection group, *n* = 104) or liver transplantation between January 2005 and December 2008 (transplantation group, *n* = 62) at Kyoto University Hospital. In the transplantation group, 34 patients fulfilled the Milan criteria for transplantation, while 28 did not. The clinicopathological characteristics of the patients are summarized in Supplemental Table 7. Tumor recurrence was investigated until patient death or the end of this study (March 31, 2014). During the follow-up time, 11 patients underwent resection of HCC metastatic region (lung, n = 6; adrenal gland, n = 2; bone, n = 1; brain, n = 1; lymph node, n = 1). Written informed consent for the use of resected tissue samples and collected blood samples was obtained from all patients in accordance with the Declaration of Helsinki, and this study was approved by the institutional review committee of our hospital (authorization number: E2126).

### Construction of the transgene vector

We generated a transgene plasmid vector that expressed enhanced green fluorescence protein (EGFP) under the control of the human SOX9 promoter. The promoter activity of the 5′-flanking region of the human SOX9 gene (from −1034 bp to +67 bp; the adenine of the ATG start codon was numbered as nucleotide 1), has been previously reported^48^. We amplified the human SOX9 promoter region from the genomic DNA of human mesenchymal stem cells by polymerase chain reaction (PCR), and subsequently ligated the promoter region with the *Bgl*II–*Hind*III-digested plasmid EGFP-1 (pEGFP1; BD Biosciences, Franklin Lakes, NJ, USA).

### Generation of transgenic HCC cell lines

Human HCC cell lines (Huh7, HLF, PLC/PRF/5, and Hep3B) were obtained from the American Type Culture Collection (ATCC, Manassas, VA, USA) or Japanese Collection of Research Bioresources Cell Bank (JCRB, Osaka, Japan). All cell lines were authenticated by short tandem repeat profiling before receipt and were propagated for less than 6 months after resuscitation. The cells were cultured at 37 °C with 5% CO_2_ in Roswell Park Memorial Institute 1640 medium (Invitrogen, Carlsbad, CA, USA) supplemented with 10% fetal bovine serum (ICN, Aurora, OH, USA), and penicillin/streptomycin (Meiji Seika, Tokyo, Japan).

The transgenic vector was transfected into cells using Lipofectamine LTX (Invitrogen), according to the manufacturer’s protocol. Stably transfected cells were selected in the presence of 200 μg/mL G418 (Sigma, St Louis, MO, USA) over 30 days. The correct transgene insertion was confirmed by PCR and immunocytochemistry.

Further details are described in the Supplemental Materials and Methods^49^,^50^,^51^,^52^,^53^,^54^.

## Additional Information

**How to cite this article**: Kawai, T. *et al*. SOX9 is a novel cancer stem cell marker surrogated by osteopontin in human hepatocellular carcinoma. *Sci. Rep.* **6**, 30489; doi: 10.1038/srep30489 (2016).

## Supplementary Material

Supplementary Information

## Figures and Tables

**Table 1:** Multivariate analysis of factors predicting postoperative prognosis.

Variable	Hazard Ratio (95% CI)	*P* value
Postoperative recurrence in the resection group		
SOX9 expression	1.71 (1.07–2.73)	0.025
Portal invasion	1.95 (1.20–3.15)	0.007
Liver cirrhosis	1.45 (0.90–2.37)	0.131
Albumin (<3.5 g/dl)	1.27 (0.68–2.37)	0.459
Postoperative survival in the resection group		
Albumin (<3.5 g/dl)	2.45 (1.32–4.55)	0.005
Portal invasion	2.11 (1.19–3.74)	0.011
Liver cirrhosis	1.88 (1.04–3.39)	0.037
Postoperative recurrence in the transplantation group		
SOX9 expression	10.3 (1.18–89.9)	0.035
Poorly differentiated	5.86 (0.82–41.8)	0.078
Portal invasion	NA	0.94
Postoperative survival in the transplantation group		
Portal invasion	5.54 (1.25–24.6)	0.024
Poorly differentiated	2.01 (0.46–8.81)	0.36

Abbreviation: SOX9, sex determining region Y-box 9; CI, confidence interval; NA, not available.

## References

[b1] Bonnet D. & Dick J. E. Human acute myeloid leukemia is organized as a hierarchy that originates from a primitive hematopoietic cell. Nat Med 3, 730–737 (1997). 9212098 10.1038/nm0797-730

[b2] Singh S. K. . Identification of a cancer stem cell in human brain tumors. Cancer Res 63, 5821–5828 (2003). 14522905

[b3] Al-Hajj M., Wicha M. S., Benito-Hernandez A., Morrison S. J. & Clarke M. F. Prospective identification of tumorigenic breast cancer cells. Proc Natl Acad Sci USA 100, 3983–3988, 10.1073/pnas.0530291100 (2003). 12629218 PMC153034

[b4] Collins A. T., Berry P. A., Hyde C., Stower M. J. & Maitland N. J. Prospective identification of tumorigenic prostate cancer stem cells. Cancer Res 65, 10946–10951, 10.1158/0008-5472.CAN-05-2018 (2005). 16322242

[b5] O’Brien C. A., Pollett A., Gallinger S. & Dick J. E. A human colon cancer cell capable of initiating tumour growth in immunodeficient mice. Nature 445, 106–110, 10.1038/nature05372 (2007). 17122772

[b6] Ricci-Vitiani L. . Identification and expansion of human colon-cancer-initiating cells. Nature 445, 111–115, 10.1038/nature05384 (2007). 17122771

[b7] Clarke M. F. . Cancer stem cells–perspectives on current status and future directions: AACR Workshop on cancer stem cells. Cancer Res 66, 9339–9344, 10.1158/0008-5472.CAN-06-3126 (2006). 16990346

[b8] Pardal R., Clarke M. F. & Morrison S. J. Applying the principles of stem-cell biology to cancer. Nat Rev Cancer 3, 895–902, 10.1038/nrc1232 (2003). 14737120

[b9] Dean M., Fojo T. & Bates S. Tumour stem cells and drug resistance. Nat Rev Cancer 5, 275–284, 10.1038/nrc1590 (2005). 15803154

[b10] Phillips T. M., McBride W. H. & Pajonk F. The response of CD24(−/low)/CD44+ breast cancer-initiating cells to radiation. J Natl Cancer Inst 98, 1777–1785, 10.1093/jnci/djj495 (2006). 17179479

[b11] Yamashita T., Budhu A., Forgues M. & Wang X. W. Activation of hepatic stem cell marker EpCAM by Wnt-beta-catenin signaling in hepatocellular carcinoma. Cancer Res 67, 10831–10839, 10.1158/0008-5472.CAN-07-0908 (2007). 18006828

[b12] Yang Z. F. . Significance of CD90+ cancer stem cells in human liver cancer. Cancer Cell 13, 153–166, 10.1016/j.ccr.2008.01.013 (2008). 18242515

[b13] Ma S. . Identification and characterization of tumorigenic liver cancer stem/progenitor cells. Gastroenterology 132, 2542–2556, 10.1053/j.gastro.2007.04.025 (2007). 17570225

[b14] Lee T. K. . CD24(+) liver tumor-initiating cells drive self-renewal and tumor initiation through STAT3-mediated NANOG regulation. Cell Stem Cell 9, 50–63, 10.1016/j.stem.2011.06.005 (2011). 21726833

[b15] Haraguchi N. . CD13 is a therapeutic target in human liver cancer stem cells. J Clin Invest 120, 3326–3339, 10.1172/JCI42550 (2010). 20697159 PMC2929722

[b16] Oikawa T. . Sal-like protein 4 (SALL4), a stem cell biomarker in liver cancers. Hepatology 57, 1469–1483, 10.1002/hep.26159 (2013). 23175232 PMC6669886

[b17] Kawai T. . Keratin 19, a Cancer Stem Cell Marker in Human Hepatocellular Carcinoma. Clin Cancer Res 21, 3081–3091, 10.1158/1078-0432.CCR-14-1936 (2015). 25820415

[b18] Lefebvre V., Dumitriu B., Penzo-Méndez A., Han Y. & Pallavi B. Control of cell fate and differentiation by Sry-related high-mobility-group box (Sox) transcription factors. Int J Biochem Cell Biol 39, 2195–2214, 10.1016/j.biocel.2007.05.019 (2007). 17625949 PMC2080623

[b19] Lincoln J., Kist R., Scherer G. & Yutzey K. E. Sox9 is required for precursor cell expansion and extracellular matrix organization during mouse heart valve development. Dev Biol 305, 120–132, 10.1016/j.ydbio.2007.02.002 (2007). 17350610 PMC1920559

[b20] Perl A. K., Kist R., Shan Z., Scherer G. & Whitsett J. A. Normal lung development and function after Sox9 inactivation in the respiratory epithelium. Genesis 41, 23–32, 10.1002/gene.20093 (2005). 15645446

[b21] Seymour P. A. . SOX9 is required for maintenance of the pancreatic progenitor cell pool. Proc Natl Acad Sci USA 104, 1865–1870, 10.1073/pnas.0609217104 (2007). 17267606 PMC1794281

[b22] Vidal V. P. . Sox9 is essential for outer root sheath differentiation and the formation of the hair stem cell compartment. Curr Biol 15, 1340–1351, 10.1016/j.cub.2005.06.064 (2005). 16085486

[b23] Poché R. A., Furuta Y., Chaboissier M. C., Schedl A. & Behringer R. R. Sox9 is expressed in mouse multipotent retinal progenitor cells and functions in Müller glial cell development. J Comp Neurol 510, 237–250, 10.1002/cne.21746 (2008). 18626943 PMC4412477

[b24] Stolt C. C. . The Sox9 transcription factor determines glial fate choice in the developing spinal cord. Genes Dev 17, 1677–1689, 10.1101/gad.259003 (2003). 12842915 PMC196138

[b25] Blache P. . SOX9 is an intestine crypt transcription factor, is regulated by the Wnt pathway, and represses the CDX2 and MUC2 genes. J Cell Biol 166, 37–47, 10.1083/jcb.200311021 (2004). 15240568 PMC2172132

[b26] Antoniou A. . Intrahepatic bile ducts develop according to a new mode of tubulogenesis regulated by the transcription factor SOX9. Gastroenterology 136, 2325–2333, 10.1053/j.gastro.2009.02.051 (2009). 19403103 PMC2743481

[b27] Kawaguchi Y. Sox9 and programming of liver and pancreatic progenitors. J Clin Invest 123, 1881–1886, 10.1172/JCI66022 (2013). 23635786 PMC3635727

[b28] Cardinale V. . Multipotent stem/progenitor cells in human biliary tree give rise to hepatocytes, cholangiocytes, and pancreatic islets. Hepatology 54, 2159–2172, 10.1002/hep.24590 (2011). 21809358

[b29] Furuyama K. . Continuous cell supply from a Sox9-expressing progenitor zone in adult liver, exocrine pancreas and intestine. Nat Genet 43, 34–41, 10.1038/ng.722 (2011). 21113154

[b30] Carpentier R. . Embryonic ductal plate cells give rise to cholangiocytes, periportal hepatocytes, and adult liver progenitor cells. Gastroenterology 141, 1432–1438, 1438.e1431-1434, 10.1053/j.gastro.2011.06.049 (2011). 21708104 PMC3494970

[b31] Liu C. . Sox9 Regulates Self-Renewal and Tumorigenicity by Promoting Symmetrical Cell Division of Cancer Stem Cells in Hepatocellular Carcinoma. Hepatology, 10.1002/hep.28509 (2016). 26910875

[b32] Vietor I., Kurzbauer R., Brosch G. & Huber L. A. TIS7 regulation of the beta-catenin/Tcf-4 target gene osteopontin (OPN) is histone deacetylase-dependent. J Biol Chem 280, 39795–39801, 10.1074/jbc.M509836200 (2005). 16204248

[b33] Pratt S. . The multidrug resistance protein 5 (ABCC5) confers resistance to 5-fluorouracil and transports its monophosphorylated metabolites. Mol Cancer Ther 4, 855–863, 10.1158/1535-7163.MCT-04-0291 (2005). 15897250

[b34] Pattabiraman D. R. & Weinberg R. A. Tackling the cancer stem cells - what challenges do they pose? Nat Rev Drug Discov 13, 497–512, 10.1038/nrd4253 (2014). 24981363 PMC4234172

[b35] Thiery J. P. Epithelial-mesenchymal transitions in tumour progression. Nat Rev Cancer 2, 442–454, 10.1038/nrc822 (2002). 12189386

[b36] Yang J. & Weinberg R. A. Epithelial-mesenchymal transition: at the crossroads of development and tumor metastasis. Dev Cell 14, 818–829, 10.1016/j.devcel.2008.05.009 (2008). 18539112

[b37] Takahashi-Yanaga F. & Kahn M. Targeting Wnt signaling: can we safely eradicate cancer stem cells? Clin Cancer Res 16, 3153–3162, 10.1158/1078-0432.CCR-09-2943 (2010). 20530697

[b38] Hanley K. P. . Ectopic SOX9 mediates extracellular matrix deposition characteristic of organ fibrosis. J Biol Chem 283, 14063–14071, 10.1074/jbc.M707390200 (2008). 18296708

[b39] Pritchett J. . Osteopontin is a novel downstream target of SOX9 with diagnostic implications for progression of liver fibrosis in humans. Hepatology 56, 1108–1116, 10.1002/hep.25758 (2012). 22488688 PMC3638324

[b40] Giannelli G., Villa E. & Lahn M. Transforming growth factor-β as a therapeutic target in hepatocellular carcinoma. Cancer Res 74, 1890–1894, 10.1158/0008-5472.CAN-14-0243 (2014). 24638984

[b41] Klaus A. & Birchmeier W. Wnt signalling and its impact on development and cancer. Nat Rev Cancer 8, 387–398, 10.1038/nrc2389 (2008). 18432252

[b42] Reya T. & Clevers H. Wnt signalling in stem cells and cancer. Nature 434, 843–850, 10.1038/nature03319 (2005). 15829953

[b43] Pez F. . Wnt signaling and hepatocarcinogenesis: molecular targets for the development of innovative anticancer drugs. J Hepatol 59, 1107–1117, 10.1016/j.jhep.2013.07.001 (2013). 23835194

[b44] Topol L., Chen W., Song H., Day T. F. & Yang Y. Sox9 inhibits Wnt signaling by promoting beta-catenin phosphorylation in the nucleus. J Biol Chem 284, 3323–3333, 10.1074/jbc.M808048200 (2009). 19047045 PMC2631972

[b45] Larsimont J. C. . Sox9 Controls Self-Renewal of Oncogene Targeted Cells and Links Tumor Initiation and Invasion. Cell Stem Cell 17, 60–73, 10.1016/j.stem.2015.05.008 (2015). 26095047

[b46] Shang S. . Identification of osteopontin as a novel marker for early hepatocellular carcinoma. Hepatology 55, 483–490, 10.1002/hep.24703 (2012). 21953299 PMC3914762

[b47] Zhou C. . Postoperative serum osteopontin level is a novel monitor for treatment response and tumor recurrence after resection of hepatitis B-related hepatocellular carcinoma. Ann Surg Oncol 20, 929–937, 10.1245/s10434-012-2749-9 (2013). 23203407

[b48] Piera-Velazquez S. . Regulation of the human SOX9 promoter by Sp1 and CREB. Exp Cell Res 313, 1069–1079, 10.1016/j.yexcr.2007.01.001 (2007). 17289023 PMC2118054

[b49] Ishii T. . *In vitro* differentiation and maturation of mouse embryonic stem cells into hepatocytes. Exp Cell Res 309, 68–77, 10.1016/j.yexcr.2005.05.028 (2005). 16009362

[b50] Ishii T. . Transplantation of embryonic stem cell-derived endodermal cells into mice with induced lethal liver damage. Stem Cells 25, 3252–3260, 10.1634/stemcells.2007-0199 (2007). 17885077

[b51] Ishii T. . Alpha-fetoprotein producing cells act as cancer progenitor cells in human cholangiocarcinoma. Cancer Lett 294, 25–34, 10.1016/j.canlet.2010.01.019 (2010). 20149523

[b52] Sasaki N. . Alpha-fetoprotein-producing pancreatic cancer cells possess cancer stem cell characteristics. Cancer Lett 308, 152–161, 10.1016/j.canlet.2011.04.023 (2011). 21616586

[b53] Yamanaka K. . Olprinone attenuates excessive shear stress through up-regulation of endothelial nitric oxide synthase in a rat excessive hepatectomy model. Liver Transpl 17, 60–69, 10.1002/lt.22189 (2011). 21254346

[b54] Hu Y. & Smyth G. K. ELDA: extreme limiting dilution analysis for comparing depleted and enriched populations in stem cell and other assays. J Immunol Methods 347, 70–78, 10.1016/j.jim.2009.06.008 (2009). 19567251

