# Supplementary material for: SOX9 is a novel cancer stem cell marker surrogated by osteopontin in human hepatocellular carcinoma
Source: Sci Rep. 2016 Jul 26;6:30489. doi: 10.1038/srep30489 (PMC4960550; doi:10.1038/srep30489)

Supplementary Information

SOX9 is a novel cancer stem cell marker surrogated by osteopontin in human hepatocellular carcinoma

Takayuki Kawai[1], Kentaro Yasuchika[1], Takamichi Ishii[1], Yuya Miyauchi[1], Hidenobu Kojima[1], Ryoya Yamaoka[1], Hokahiro Katayama[1], Elena Yukie Yoshitoshi[1, 2], Satoshi Ogiso[1], Sadahiko Kita[1], Katsutaro Yasuda[1, 2], Ken Fukumitsu[1], Junji Komori[1], Etsuro Hatano[1], Yoshiya Kawaguchi[1, 2], and Shinji Uemoto[1]

[1]Department of Surgery, Graduate School of Medicine, Kyoto University, Kyoto, Japan;

[2]Center for iPS Cell Research and Application (CiRA), Kyoto University, Kyoto, Japan

Supplemental Materials and methods

Immunohistochemistry

We fixed liver specimens obtained from HCC patients and stained as previously described.[49, 50] Anti-human SOX9 rabbit antibodies (Millipore, Billerica, MA, USA) diluted at 1:200 and anti-human OPN goat antibodies (R&D systems, Minneapolis, MN, USA) diluted at 1:50 were used as the primary antibodies. Alexa 488-conjugated donkey

anti-rabbit IgG (Molecular Probes, Eugene, OR, USA) for SOX9 staining and Alexa 594-conjugated goat anti-goat IgG (Molecular Probes) for OPN staining were used as the secondary antibodies. All secondary antibodies were diluted at 1:500. Two investigators (T.K. and K.Y.) independently evaluated the slides.

Immunocytochemistry of cultured cells

We fixed the cultured cells and stained as previously described.[49, 50] Anti-human SOX9 rabbit antibodies (Millipore), anti GFP chicken antibodies (Invitrogen), and anti activated beta-catenin mouse antibodies (Millipore) were used as the primary antibodies. All primary antibodies were diluted at 1:200. Alexa 594-conjugated donkey anti-rabbit IgG (Molecular Probes) for SOX9 staining, Alexa 488-conjugated goat anti-chicken IgG (Molecular Probes) for GFP staining, and Alexa 594-conjugated rabbit anti-mouse IgG (Molecular Probes) for activated beta-catenin staining were used as the secondary antibodies. All secondary antibodies were diluted at 1:500. The stained cells were covered with Vectashield mounting medium with DAPI (Vector Laboratories).

PCR, quantitative PCR, reverse transcription-PCR (RT-PCR), and quantitative RT-PCR (qRT-PCR)

The total RNA was extracted with RNeasy Mini Kit (Qiagen, Chatsworth, CA, USA) and RNase-free DNAse (Qiagen). The genomic DNA was extracted with QuickGene-SP Kit (FUJIFILM, Tokyo, Japan). The Omniscript Reverse Transcription Kit (Qiagen) was used according to the manufacturer's protocol to reverse transcribe 1 μg total RNA into cDNA. Primers were generated for the following genes: SOX9, SOX9 promoter, plasmid EGFP-1 (pEGFP1), epithelial cell adhesion molecule (EpCAM), cluster of differentiation (CD) 90, CD133, CD24, CD13, sal-like protein 4 (SALL4), keratin 19 (K19), transforming growth factor beta receptor 1 (TGFbR1), TGFbR2, snail1, E-cadherin, vimentin, multidrug-resistance protein-5 (MRP5), OPN, cyclin D1 and actin-beta. Their sequences are summarized in Supplemental Table 8. PCR and RT-PCR assays were performed as previously described.[51] We performed qPCR and qRT-PCR assays using SYBR-green PCR Master Mix (Applied Biosystems, Foster City, CA, USA) on the ABI 7500 system (Applied Biosystems). Each target was run in triplicate, and expression levels were normalized to those of actin-beta.

Flow cytometry and single-cell culture analysis

We prepared the cultured cells as described previously.[17, 51, 52] Dead cells were eliminated using 7-amino-actinomycin D (Beckman Coulter, Brea, CA, USA) staining.

We performed single-cell culture analyses as previously described.[14, 47, 48] The individual isolated cells were each sorted into 96-well culture plates using FACSAria (BD Biosciences). We used a light microscope 10–16h after cell sorting to confirm that each well contained only one cell. Following cell expansion after isolation of each clone, we subjected the cells to flow cytometry.

Cell proliferation assay, anchorage-independent growth assay, and sphere-forming assay

We inoculated the isolated $EGFP^+$ and $EGFP^-$ cells differentiated from one $EGFP^+$ cell at a density of 1×10^3 cells per well in 96-well culture plates, which were then allowed to grow for 7 days. The cell numbers were determined using the 3-(4,5-dimethylthiazol-2-yl)-5-(3-carboxymethoxyphenyl)-2-(4-sulfophenyl)-2H-tetrazorium, inner salt (MTS) assay (Cell Titer 96 Aqueous One Solution Reagent, Promega), according to the manufacturer's protocol. After 1 h of incubation, the absorbance value was measured using a plate reader at 490 nm.

To examine the anchorage-independent growth, 1×10^4 $EGFP^+$ and $EGFP^-$ cells were suspended in 2.0 mL of 0.3% agar (Wako) supplemented with culture medium. The

cell suspension was layered over the bottom layer of 2.0 mL of 0.6% agar. We counted the colonies 14 days after cell sorting.

To investigate the ability to form cell spheres, 1×10^5 $EGFP^+$ and $EGFP^-$ were seeded in 6-well ultra-low attachment plates (Corning Inc., NY, USA) in serum-free medium. We observed the spheres 5 days after cell sorting.

Reagents and drug resistance assay

5-Fluorouracil (5-FU), TGFb1, and GSK-3b inhibitor CHIR99021 were purchased from Wako and was diluted directly with RPMI-1640 to the desired concentration. TGFb receptor 1 (TGFbR1) inhibitor LY2157299 was obtained from Axon Medchem (Groningen, NL). TGFbR1/R2 inhibitor LY2109761 was purchased from Selleck (Houston, TX, USA). Compounds were dissolved in 100% dimethyl sulfoxide (DMSO; Sigma) and diluted with RPMI-1640 or saline to the desired concentration with a final DMSO concentration of under 0.5%. We investigated the 5-FU resistance of the cells. $EGFP^+$ and $EGFP^-$ cells were cultured with 5-FU for 96h at concentrations of 1×10^{-9}, 1×10^{-7}, 1×10^{-5}, 1×10^{-3}, and 1×10^{-1} M. After the culture, MTS assays were performed to determine the half-maximal inhibitory concentrations (IC50).

Xenotransplantation

Male 6 to 10-week-old non-obese diabetic/severe combined immunodeficiency (NOD/SCID) mice (Charles River Laboratories, Inc., Kanagawa, Japan) were used as recipients for xenotransplantation. The isolated $EGFP^+$ or $EGFP^-$ cells (1.0×10^3 or 1.0×10^4 cells) derived from a single $EGFP^+$ cell were suspended in 200 μL of a mixture of serum-free medium and Matrigel (BD Biosciences) (1:1 volume). The mixture was injected subcutaneously through a 26-gauge needle into the right and left dorsal areas of anesthetized NOD/SCID mice. We monitored tumor formation and tumor size twice a week, and dissected out the tumors 11 weeks after engraftment.

To investigate the differentiation ability *in vivo*, we performed serial transplantation. Tumors generated from single-cell-derived $EGFP^+$ or $EGFP^-$ cells were harvested, and then digested with collagenase solution for 30 min at 37°C. After rinsing the tumors in phosphate-buffered saline (PBS), we analyzed and sorted the cells using the FACSAria. Prior to the second transplantation, the harvested cells were cultured in G418-containing medium for at least 7 days to eliminate cells that originated from the host mice. Thereafter, we sorted these cells according to the EGFP fluorescence, and transplanted 1×10^4 $EGFP^+$ or $EGFP^-$ cells into NOD/SCID mice in the same way as in the first transplantation.

All animal experimental procedures were performed according to the Animal Protection Guidelines of Kyoto University.

Wound healing assay and migration assay

Wound healing assays were used to assess capacity for cell motility. We seeded the isolated $EGFP^+$ and $EGFP^-$ cells differentiated from one $EGFP^+$ cell at a density of 1×10^6 cells per well in 35-mm culture dishes. On reaching full confluency, the cell layer was scratched with a 10-μL plastic tip and then cultured with low serum (2% fetal bovine serum) culture medium and 10 ng/ml TGFb. Micrographs were taken at 24 h after the scratch.

For migration assays, 8-μm-pore 24-well cell culture plates (Corning Inc.) coated with type I collagen were used. We plated 2.5×10^4 $EGFP^+$ and $EGFP^-$ cells in the upper chamber with serum-free medium and 10 ng/ml TGFb; in the lower chamber, normal culture medium containing 10% fetal bovine serum was added. After 48 h of incubation, the cells on the upper surface of the membrane were removed, and the cells on the lower surface were fixed with 4% paraformaldehyde and stained using the Diff-Quick staining kit (Sysmex, Kobe, Japan).

Western blot analysis

Western blot analysis was performed as previously reported.[53] Primary antibodies recognizing SOX9 (Millipore), OPN (R&D), phospho-smad2 (pSmad2) (Ser465/467, #3108; Cell Signaling, Tokyo, Japan), Smad2 (#5339, Cell Signaling), snail1 (#3879, Cell Signaling), E-cadherin (#14472, Cell Signaling), vimentin (#5741, Cell Signaling), and glyceraldehyde-3-phosphate dehydrogenase (GAPDH) (sc-25778, Santa Cruz Biotechnology Inc., Santa Cruz, CA) were used at 1:1000 dilution. HRP-conjugated bovine anti-rabbit IgG (Molecular Probes) for SOX9, pSmad2, Smad2, snail1, vimentin, and GAPDH staining, HRP-conjugated bovine anti-mouse IgG (Molecular Probes) for E-cadherin staining, and HRP-conjugated bovine anti-goat IgG (Molecular Probes) for OPN staining were used as the secondary antibodies. All secondary antibodies were diluted at 1:2000.

SOX9 knockdown and overexpression

For SOX9 knockdown experiments, we transfected SOX9-siRNA (s13306 or s13307, Invitrogen) or control-siRNA (#4390843, Invitrogen) into HCC cells using Lipofectamine LTX (Invitrogen) according to the manufacturer's protocol. The final concentrations were 10nM. SOX9 expression was significantly downregulated by both SOX9 siRNAs

(Supplemental Fig. 5A). Because the same results were acquired with both siRNAs in all SOX9 knockdown experiments, SOX9-siRNA (s13307) was shown as representative data. For SOX9 overexpression experiments, we transfected SOX9 expression vector (SC321884, OriGene, Rockville, USA) or mock vector (PS100020, OriGene) into HCC cell lines using Lipofectamine LTX (Invitrogen) according to the manufacturer's protocol. SOX9 overexpression was confirmed by qRT-PCR analyses (Supplemental Fig. 5C). For western blot analysis, HCC cells were harvested 48 h post-transfection in SOX9 knockdown/overexpression experiments.

OPN knockdown and overexpression

For OPN knockdown experiments, we transfected OPN-siRNA (s13376 or s13377, Invitrogen) or control-siRNA (#4390843, Invitrogen) into HCC cells using Lipofectamine LTX (Invitrogen) according to the manufacturer's protocol. The final concentrations were 10nM. OPN expression was significantly downregulated by both OPN siRNAs (Supplemental Fig. 5B). Because the same results were acquired with both siRNAs in all OPN knockdown experiments, OPN-siRNA (s13376) was shown as representative data. For OPN overexpression experiments, we amplified the human OPN open reading frame by RT-PCR, and then ligated the open reading frame with Sgf1–RsrII-digested

plasmid CMV6-AC (PS100020, OriGene). We transfected this OPN expression vector or mock vector (PS100020, OriGene) into HCC cell lines using Lipofectamine LTX (Invitrogen) according to the manufacturer's protocol. OPN overexpression was confirmed by qRT-PCR analyses (Supplemental Fig. 5D). For western blot analysis, HCC cells were harvested 48 h post-transfection in OPN knockdown/overexpression experiments.

T-cell factor/lymphoid enhancer factor (TCF/LEF) luciferase assay

To evaluate the activation of Wnt/beta-catenin pathway in HCC cells, we performed TCF/LEF luciferase assay in addition to immunocytochemistry of activated beta-catenin. Cignal TCF/LEF Reporter Assay Kit (Qiagen) and Dual-Glo™ Luciferase Assay System (Promega) were used according to the manufacturer's protocols.

Enzyme-linked immunosorbent assays (ELISA)

To measure the serum OPN level of HCC patients, we performed ELISA. Blood samples were collected and stored at 4°C for 1 h. Serum was separated by centrifugation (10,000 g for 10 min) at 4°C, and then stored at −80°C until analysis. We quantitated serum OPN levels, respectively, with the human OPN ELISA kits (Millipore) according

to the manufacture's protocols. The OPN concentration was calculated by a standard curve. Each serum sample was tested in duplicate, and the results were averaged to maintain reliability.

Statistical analysis

The statistical analyses were performed using SPSS version 17.0 (SPSS Inc., IL, USA) and GraphPad Prism software version 5.0 (GraphPad Software Inc., San Diego, CA, USA). Student's *t*-test, *F*-test, Mann-Whitney *U* test, Fisher's exact test or chi-squared test, log-rank test, and repeated-measures analyses of variances were used for assessment. The tumor initiating frequency in xenotransplantation was calculated by extreme limiting dilution analysis. [54] The mean ± SD of three or more independent experiments is reported.

Recurrence-free survival (RFS) and overall survival (OS) after the operation were calculated using the Kaplan-Meier method and analyzed with the log-rank test. Significant variables from the univariate analysis were entered in the multivariate analysis using a Cox regression model with forward stepwise selection. We plotted receiver operating characteristics (ROC) curve for serum OPN level and HCC tumor markers, and calculated area under the ROC curve (AUC). The optimal cutoff value of

OPN was calculated using the maximum sum of sensitivity and specificity as well as using the minimum distance to the top-left corner of the ROC curve. Statistical significance was defined as $P < 0.05$.

Supplemental Table 1

Tumor incidence in initial/serial transplantation

	initial Tx			serial Tx
	1.0×10^4 cells	1.0×10^3 cells	1.0×10^2 cells	1.0×10^4 cells
Huh7 $SOX9^+$	8/8 (100%)	4/5 (80%)	1/3 (33.3%)	7/8 (87.5%)
Huh7 $SOX9^-$	3/8 (37.5%)	0/5 (0%)	0/3 (0%)	1/8 (12.5%)
HLF $SOX9^+$	8/8 (100%)	5/5 (100%)	2/3 (66.7%)	8/8 (100%)
HLF $SOX9^-$	3/8 (37.5%)	1/5 (20%)	0/3 (0%)	2/8 (25%)
PLC/PRF/5 $SOX9^+$	7/8 (87.5%)	5/5 (100%)	1/3 (33.3%)	6/7 (85.7%)
PLC/PRF/5 $SOX9^-$	3/8 (37.5%)	0/5 (0%)	0/3 (0%)	1/7 (14.3%)
Hep3B $SOX9^+$	6/8 (75%)	4/5 (80%)	1/3 (33.3%)	5/6 (83.3%)
Hep3B $SOX9^-$	3/8 (37.5%)	0/5 (0%)	0/3 (0%)	0/6 (0%)

Abbreviation: SOX9, sex determining region Y-box 9; Tx, transplantation.

Supplemental Table 2

The tumor initiating frequency in HCC cells

	CSC frequency			*P* value
	Lower	Estimate	Upper	
Huh7 $SOX9^+$	1473	531	192	$P < 0.01$
Huh7 $SOX9^-$	71609	23074	7435	
HLF $SOX9^+$	389	92	22	$P < 0.01$
HLF $SOX9^-$	47030	17086	6207	
PLC/PRF/5 $SOX9^+$	5780	2010	699	$P < 0.01$
PLC/PRF/5 $SOX9^-$	71609	23074	7435	
Hep3B $SOX9^+$	8641	3742	1621	$P < 0.01$
Hep3B $SOX9^-$	71609	23074	7435	

Abbreviation: SOX9, sex determining region Y-box 9; CSC, cancer stem cell.

Supplemental Table 3

SOX9 and OPN expression of 11 patients with a resection of HCC metastatic region

Patient	primary HCC	metastatic HCC	metastatic organ
#1	SOX9−OPN−	SOX9−OPN−	Adrenal
#2	SOX9−OPN−	SOX9+OPN+	Lung
#3	SOX9−OPN−	SOX9+OPN+	Bone
#4	SOX9−OPN−	SOX9+OPN+	Lung

#5	SOX9−OPN+	SOX9+OPN+	Lung
#6	SOX9+OPN+	SOX9+OPN+	Lung
#7	SOX9+OPN+	SOX9+OPN+	Brain
#8	SOX9−OPN−	SOX9+OPN+	Adrenal
#9	SOX9+OPN+	SOX9+OPN+	Lymph node
#10	SOX9−OPN+	SOX9+OPN+	Lung
#11	SOX9−OPN−	SOX9−OPN−	Lung

Abbreviation: SOX9, sex determining region Y-box 9; OPN, osteopontin.

Supplemental Table 4

Univariate analysis with respect to outcome in the resection group

Recurrence-free survival				
Factors	Number of patients	Recurrence-free survival days (median, 95% CI)	*P* value	Hazard Ratio (median, 95% CI)
SOX9 expression				
Positive	37	378 (270-485)	0.027	1.74

Negative	67	765 (460-1069)		(1.06-2.86)
Age (years)				
≧65	65	558 (332-784)	0.108	1.45
＜65	39	688 (33-1343)		(0.92-2.28)
Gender				
Male	83	644 (469-859)	0.131	0.63
Female	21	351 (276-426)		(0.35-1.15)
AST activity (IU/L)				
≧40	66	568 (269-867)	0.318	1.26
＜40	38	664 (406-922)		(0.8-1.98)
ALT activity (IU/L)				
≧40	57	576 (365-787)	0.375	1.22
＜40	47	640 (359-921)		(0.78-1.91)
Total billirubin (mg/dl)				
＞1.0	27	765 (213-1317)	0.618	1.15
≦1.0	77	558 (316-800)		(0.69-1.89)
Albumin (g/dl)				

＜3.5	24	231 (72-390)	0.003	2.74
≧3.5	80	674 (453-894)		(1.41-5.32)
Platelet count				
($10^4/mm^3$)				
＜10	27	375 (19-731)	0.13	1.51
≧10	77	640 (521-759)		(0.89-2.58)
AFP (ng/ml)				
≧20	55	568 (287-849)	0.388	1.22
＜20	49	674 (391-957)		(0.78-1.90)
PIVKA-II (mAU/ml)				
≧40	71	473 (210-736)	0.368	1.24
＜40	33	653 (529-777)		(0.78-1.97)
Hepatitis B infection				
Present	13	375 (266-484)	0.907	0.96
Absent	91	653 (503-803)		(0.48-1.91)
Hepatitis C infection				
Present	64	568 (279-857)	0.308	1.26
Absent	40	674 (348-1000)		(0.81-1.98)

Tumor size (cm)				
≧5cm	47	473 (64-882)	0.635	1.12
＜5cm	57	653 (514-792)		(0.71-1.76)
Tumor number				
Multiple	33	473 (84-862)	0.231	1.34
Single	71	586 (463-709)		(0.83-2.17)
Tumor differentiation				
Poor	18	716 (602-830)	0.782	0.92
Others	80	653 (520-786)		(0.49-1.71)
Portal Invasion				
Positive	36	267 (132-402)	0.007	2.08
Negative	68	800 (418-1182)		(1.23-3.54)
Liver cirrhosis				
F4	33	418 (172-663)	0.027	1.79
Others	71	688 (428-948)		(1.07-3.00)
Type of resection				
Anatomic	75	568 (287-849)	0.616	1.13

Partial	29	674 (387-961)		(0.69-1.83)

Overall survival				
Factors	Number of patients	Overall survival days (median, 95%CI)	*P* value	Hazard Ratio (median, 95% CI)
SOX9 expression				
Positive	37	1929 (1018-2840)	0.12	1.62
Negative	67	2198 (1929-2467)		(0.89-2.94)
Age (years)				
≧65	64	2093 (1574-2611)	0.05	1.76
＜65	40	NA		(0.99-3.09)
Gender				
Male	83	1929 (1085-2773)	0.131	0.85
Female	21	2198 (1936-2460)		(0.43-1.72)
AST activity (IU/L)				
≧40	63	2093 (1812-2374)	0.341	1.32
＜40	41	2286		(0.75-2.34)

ALT activity (IU/L)				
≧40	57	2162 (1901-2423)	0.872	1.05
<40	47	2248 (1578-2918)		(0.59-1.83)
Total billirubin (mg/dl)				
>1.0	27	2198 (2071-2325)	0.971	1.01
≦1.0	77	2093		(0.54-1.88)
Albumin (g/dl)				
<3.5	24	974 (523-1425)	0.003	4.34
≧3.5	80	2286		(2.04-9.25)
Platelet count ($10^4/mm^3$)				
<10	27	1977 (1089-2865)	0.155	1.61
≧10	77	2286		(0.84-3.09)
AFP (ng/ml)				
≧20	55	2093 (1806-2380)	0.15	1.53
<20	49	NA		(0.88-2.65)
PIVKA-II (mAU/ml)				

≧40	71	2162	0.845	1.06
＜40	33	2198		(0.59-1.91)
Hepatitis B infection				
Present	13	NA	0.908	0.93
Absent	91	2198 (1907-2489)		(0.41-2.15)
Hepatitis C infection				
Present	64	NA	0.119	1.57
Absent	40	1960 (1605-2315)		(0.89-2.75)
Tumor size (cm)				
≧5cm	78	1901 (1019-2783)	0.09	1.63
＜5cm	26	2248 (2036-2461)		(0.93-2.87)
Tumor number				
Multiple	33	2248	0.157	0.66
Single	71	1977 (1501-2453)		(0.37-1.18)
Tumor differentiation				
Poor	18	1095 (NA-2670)	0.171	1.77
Others	80	2248 (2053-2443)		(0.78-4.03)
Portal invasion				

Positive	36	1049 (864-1234)	0.007	2.61
Negative	68	2286		(1.38-4.92)
Liver cirrhosis				
F4	33	1498 (415-2581)	0.004	2.53
Others	71	NA		(1.35-4.71)
Type of resection				
Anatomic	75	2198 (1403-2993)	0.228	1.44
Partial	29	2248 (2032-2464)		(0.79-2.62)

Abbreviation: SOX9, sex determining region Y-box 9; AST, asparatate aminotransferase; ALT, alanine aminotransferase; AFP, alpha-fetoprotein; PIVKA-II, protein induced by vitamin K absence or antagonists-II; NA, not available; CI, confidence interval.

Supplemental Table 5

Univariate analysis with respect to outcome in the transplantation group

Recurrence-free survival				
Factors	Number of	Recurrence-free survival days	*P* value	Hazard Ratio (median, 95%

	patients	(median, 95% CI)		CI)
SOX9 expression				
Positive	20	2344 (1894-2795)	0.044	6.1
Negative	42	2746 (2624-2869)		(1.05-35.4)
Age (years)				
≧60	18	2571 (2225-2917)	0.758	1.33
＜60	44	2651 (2453-2849)		(0.22-7.98)
Gender				
Male	47	NA	0.151	3.83
Female	15	NA		(0.61-24.0)
AFP (ng/ml)				
≧20	37	NA	0.65	1.45
＜20	25	NA		(0.29-7.36)
PIVKA-II (mAU/ml)				
≧40	30	NA	0.08	4.11
＜40	32	NA		(0.83-20.4)
Hepatitis B infection				

Present	42	2655 (2479-2850)	0.34	0.64
Absent	20	2518 (2152-2885)		(0.06-1.58)
Hepatitis C infection				
Present	24	2484 (2140-2828)	0.156	2.31
Absent	38	2706 (2533-2880)		(0.41-12.9)
Milan criteria				
over	28	NA	0.004	10.5
within	34	NA		(2.07-52.8)
Tumor differentiation				
Poor	54	2736 (2600-2872)	0.003	49.1
Others	8	1744 (986-2501)		(3.89-618.8)
Portal Invasion				
Positive	44	NA	<0.001	55.5
Negative	18	NA		(8.71-354.4)

Overall survival				
Factors	Number of	Overall survival days (median, 95%CI)	*P* value	Hazard Ratio (median, 95%

	patients			CI)
SOX9 expression				
Positive	20	2407 (2007-2806)	0.29	2.19
Negative	42	2634 (2463-2805)		(0.51-9.33)
Age (years)				
≧60	18	2583 (2254-2913)	0.863	0.87
＜60	44	2570 (2365-2775)		(0.19-4.04)
Gender				
Male	47	2537 (2396-2679)	0.321	2.14
Female	15	2508 (2283-2733)		(0.48-9.59)
AFP (ng/ml)				
≧20	37	NA	0.96	0.97
＜20	25	NA		(0.26-3.62)
PIVKA-II (mAU/ml)				
≧40	30	NA	0.27	2.09
＜40	32	NA		(0.56-7.72)
Hepatitis B infection				

Present	42	2600 (2405-2796)	0.479	1.66
Absent	20	2487 (2137-2837)		(0.41-6.65)
Hepatitis C infection				
Present	24	2459 (2127-2390)	0.344	0.53
Absent	38	2632 (2441-2823)		(0.14-2.01)
Milan criteria				
over	28	2120 (1814-2426)	0.029	4.37
within	34	2770 (2654-2885)		(1.16-16.4)
Tumor differentiation				
Poor	54	2657 (2505-2810)	0.03	10.3
Others	8	1786 (1067-2504)		(1.26-84.8)
Portal Invasion				
Positive	44	2744 (2617-2871)	0.002	11.6
Negative	18	2042 (1585-2500)		(2.46-55.1)

Abbreviation: SOX9, sex determining region Y-box 9; AFP, alpha-fetoprotein; PIVKA-II, protein induced by vitamin K absence or antagonists-II; NA, not available; CI, confidence interval.

Supplemental Table 6

Sensitivity and Specificity of OPN, AFP, and PIVKA-II for the evaluation of SOX9 expression in the resection group

Variable (optimal or clinical cutoff value)	Sensitivity (%) (95% CI)	Specificity (%) (95% CI)
OPN (100 ng/ml)	93 (77–99)	75 (59–87)
AFP (20 ng/ml)	50 (31–69)	48 (32–64)
PIVKA-II (40 mAU/ml)	43 (24–63)	70 (53–83)

Abbreviation: OPN, osteopontin; AFP, alpha-fetoprotein; PIVKA-II, protein induced by vitamin K absence 2; CI, confidence interval.

Supplemental Table 7

Clinicopathological findings of HCC patients

Resection group	SOX9 expression		
	Positive (n=37)	Negative (n=67)	P value
Age (years, mean ± SD)	65.5 ± 9.5	67.3 ± 8.3	0.575

Gender (male/female)	30/7	53/14	0.988
Total bilirubin (> 1.0 mg/dl)	10	17	0.746
Albumin (< 3.5 g/dl)	11	13	0.374
Platelet (< 10.0×10^4/μl)	9	17	0.836
AFP (≧ 20 ng/ml)	24	35	0.301
PIVKA-II (≧ 40 mAU/ml)	25	46	0.909
HBs Ag positive	7	6	0.126
HCV Ab positive	23	42	0.923
Tumor size (> 5 cm)	12	29	0.278
Tumor differentiation			0.396
Well	7	16	
Moderate	22	36	
Poorly	5	12	
Unknown	3	3	
UICC Stage (III-IV/I-II)	21/16	37/30	0.880
Serosal invasion	4	10	0.347
Portal invasion	13	23	0.934
Liver cirrhosis	11	22	0.745

Type of operation			0.755
Anatomic resection	25	49	
Limited resection	12	18	

Transplantation group	SOX9 expression		
	Positive (n=20)	Negative (n=42)	*P* value
Age (years, mean ± SD)	56.9 ± 6.1	57.8 ± 4.6	0.523
Gender (male/female)	18/2	29/13	0.072
Laboratory data			
AFP (≧ 20 ng/ml)	12	25	0.971
PIVKA (≧ 40 mAU/ml)	12	18	0.279
HBs Ag positive	8	12	0.368
HCV Ab positive	12	26	0.886
Pathology			
Tumor size (> 5 cm)	2	1	0.191
Tumor differentiation			0.638
Poorly	2	6	

Others	18	36	
Portal invasion	8	10	0.189
Transplantation criteria			
within Milan	8	26	0.105

Abbreviation: AFP, alpha-fetoprotein; PIVKA-II, protein induced by vitamin K absence or antagonists-II; HBs Ag, hepatitis B antigen; HCV Ab, hepatitis C antibody; UICC, union for international cancer control.

Supplemental Table 8

Primer sequences for PCR, RT-PCR, qPCR, and qRT-PCR

Gene	Sense primer	Antisense primer
SOX9	GCGTATGAATCTCCTGGACC	GTCCTCCTCGCTCTCCTTCT
SOX9 promoter	GTGGAGCGTTTTGTCTGC	TGAAACTGGCGAGTCTCC
EpCAM	AATGTGTGTGCGTGGGA	TTCAAGATTGGTAAAGCCAGT
CD90	ATGAACCTGGCCATCAGCATCGC	CAGGCTGAACTCGTACTGGA
CD133	AAGGCATATGAATCCAAAATTGA	CCACCAGAGGCATCAGAATAA
CD24	ATGGGCAGAGCAATGGTG	TGGAATAAATCTGCGTGGGTA

CD13	CATCCATCAGAGATGGCAGAC	TGCTGAAGAGATCGTTCTGG
SALL4	TGCAGCAGTTGGTGGAGAAC	TCGGTGGCAAATGAGACATTC
K19	TTTGAGACGGAACAGGCTCT	TCAGTAACCTCGGACCTGCT
pEGFP1	GTTATTACTAGCGCTACCGGACTC	CGGCCATGATATAGACGTTG
TGFbR1	TGCTGCAATCAGGACCATTG	TCCTCTTCATTTGGCACTCG
TGFbR2	TGCTCACCTCCACAGTGATC	TCTGGAGCCATGTATCTTGC
snail1	ACCACTATGCCGCGCTCTT	GGTCGTAGGGCTGCTGGAA
E-cadherin	CGACCCAACCCAAGAATCTA	TTCACAGTCACACACGCTGA
vimentin	GGCTCAGATTCAGGAACAGC	GCCTCAGAGAGGTCAGCAAA
MRP5	CACCATCCACGCCTACAATAAA	CACCGCATCGCACACGTA
OPN	AGTTTCGCAGACCTGACATCCAGT	TTCATAACTGTCCTTCCCACGGCT
OPN ORF	CCGACCAAGGAAAACTCACT	CTCCTTTTAATTGACCTCAG
cyclin D1	CTGGAGGTCTGCGAGGAACA	CCTTCATCTTAGAGGCCACGAA
ACTb	TCATGAAGATCCTCACCGAG	TTGCCAATGGTGATGACCTG

Abbreviation: SOX9, sex determining region Y-box 9; pEGFP1, plasmid EGFP-1; EpCAM; epithelial cell adhesion molecule; CD90, cluster of differentiation 90; SALL4, sal-like protein 4; K19, keratin 19; TGFbR1, TGF-beta receptor 1; TGFbR2, TGF-beta receptor 2; MRP5, multidrug resistance protein 5; OPN, osteopontin; ORF, open reading

frame; ACTb, actin-beta.

Supplemental Figure legends

Supplemental Fig. 1 EGFP-marking of the SOX9$^+$ cells in human HCC cell lines. (A) RT-PCR analysis of SOX9 in the four HCC cell lines. (B) Phase-contrast images of the stable transfectants confirmed successful transfection of SOX9-EGFP transgene. Scale bar represents 100 μm. (C) Immunocytochemistry of stable transfectants showed the co-localization of SOX9 and GFP expression in SOX9$^+$ cells (left panel). The ratios of SOX9$^+$EGFP$^+$ cells to all SOX9$^+$ cells were > 95% (right panel). Data are shown as the mean ± SD. (D) FACS analyses of stable transfectants. (E) qPCR analyses of pEGFP1 gene in the genomic DNA of sorted EGFP$^+$ and EGFP$^-$ cells (Student's *t*-test, n.s.; not significant). Data are shown as the mean ± SD. (F) qRT-PCR analyses of EpCAM, CD90, CD133, CD24, CD13, and K19 in SOX9$^+$ and SOX9$^-$ cells (Student's *t*-test, * $P < 0.05$). Data are shown as the mean ± SD. Note that previously reported HCC-CSC markers were expressed more in SOX9$^+$ populations.

Supplemental Fig. 2 Cancer stem cell properties of SOX9$^+$ HLF, PLC/PRF/5, and Hep3B cells *in vitro*. (A) Single-cell culture of SOX9$^+$ and SOX9$^-$ cells. In all three cell lines,

FACS analyses revealed that isolated SOX9-EGFP$^+$ cell differentiated both EGFP$^+$ and EGFP$^-$ cell fraction, whereas isolated SOX9-EGFP$^-$ cell only to SOX9$^-$ cell fraction. (B) Cell proliferation assays showed SOX9$^+$ cells proliferate more than SOX9$^-$ cells (repeated-measures ANOVA, ** $P < 0.01$). (C) Microscopic appearance and the colony numbers in the anchorage-independent growth assay (Student's *t*-test, * $P < 0.05$). (D) Phase-contrast images in the sphere-forming assay. (E) IC_{50} of 5-FU in SOX9$^+$ and SOX9$^-$ cells (left panel, *F*-test, * $P < 0.05$) and qRT-PCR analyses of MRP5 in SOX9$^+$ and SOX9$^-$ cells (right panel, Student's *t*-test, * $P < 0.05$).

Supplemental Fig. 3 Cancer stem cell properties of SOX9$^+$ HLF, PLC/PRF/5, and Hep3B cells *in vivo*. (A) Xenotransplantation of SOX9$^+$ and SOX9$^-$ cells in NOD/SCID mice, the formed tumor, and the tumor incidence. Note the higher incidence of tumor formation by SOX9$^+$ cell transplantation (log-rank test, HLF; ** $P < 0.01$, PLC/PRF/5; * $P < 0.05$, Hep3B; $P = 0.059$). (B) Development of formed tumor from transplanted SOX9$^+$ or SOX9$^-$ cells (repeated-measures ANOVA, HLF; $P = 0.08$, PLC/PRF/5; $P = 0.10$, Hep3B; $P = 0.05$). Data are shown as the mean ± SD. (C) Immunohistochemical analysis of tumor. Note that SOX9$^+$ and SOX9$^-$ cells detected in SOX9$^+$ cell-driven tumor. Scale bar represents 100 μm. (D) FACS analyses of dissociated tumor cells confirmed bi-potent

differentiation ability conserved in serial transplantation of $SOX9^+$.

Supplemental Fig. 4 Functional rescue experiments of TGFb/Smad signaling in $SOX9^-$ Huh7 cells. (A) Western blot analysis showed pSmad2 upregulation in $SOX9^-$ Huh7 cells by high dose TGFb1 stimulation (30 ng/ml for 24 h). (B) Upon high dose TGFb1 stimulation (30 ng/ml), $SOX9^-$ Huh7 cells showed the superior sphere-forming ability (left panel) and proliferation ability (right panel) compared to the control $SOX9^-$ Huh7 cells without TGFb1 stimulation (repeated-measures ANOVA, * $P < 0.05$).

Supplemental Fig. 5 Functional rescue experiments of Wnt/beta-catenin pathway in $SOX9^-$ HLF cells. (A) CHIR99021-stimulated $SOX9^-$ HLF cells showed significantly higher TCF/LEF activity and cyclin D1 expression compared to the control $SOX9^-$ HLF cells without CHIR99021 stimulation (Student's *t*-test, * $P < 0.05$). (B) CHIR99021-stimulated $SOX9^-$ HLF cells showed superior sphere-forming ability (left panel) and proliferation (right panel) ability compared to the control $SOX9^-$ HLF cells without CHIR99021 stimulation (repeated-measures ANOVA, * $P < 0.05$).

Supplemental Fig. 6 Correlation of SOX9 and OPN expressions in human HCC. (A)

Double staining of SOX9 and OPN in primary HCC (left panels) and in HCC metastatic lesions (right panels). Scale bar represents 100 μm. (B) Correlation between SOX9 and OPN expression in the primary and metastatic HCC (Fisher's exact test, ** $P < 0.01$).

Supplemental Fig. 7 Gain/loss of SOX9 or OPN functions in HCC cells. (A) qRT-PCR analyses confirmed successful knockdown of SOX9 expression by siRNA (KD-SOX9). (B) Efficient reduction of OPN expression by siRNA (KD-OPN) revealed by qRT-PCR. (C) qRT-PCR analyses of SOX9 in the cells transfected with control-mock-vector or CMV promoter-driven SOX9-expression-vector. (D) Successful OPN overexpression by OPN-expression-vector. Data are shown as the mean ± SD (Student's *t*-test, * $P < 0.05$, KD-NC; control knockdown, KD-SOX9; SOX9 knockdown, KD-OPN; OPN knockdown, EX-NC; control overexpression, EX-SOX9; SOX9 overexpression, EX-OPN; OPN overexpression).

Supplemental Figure 1

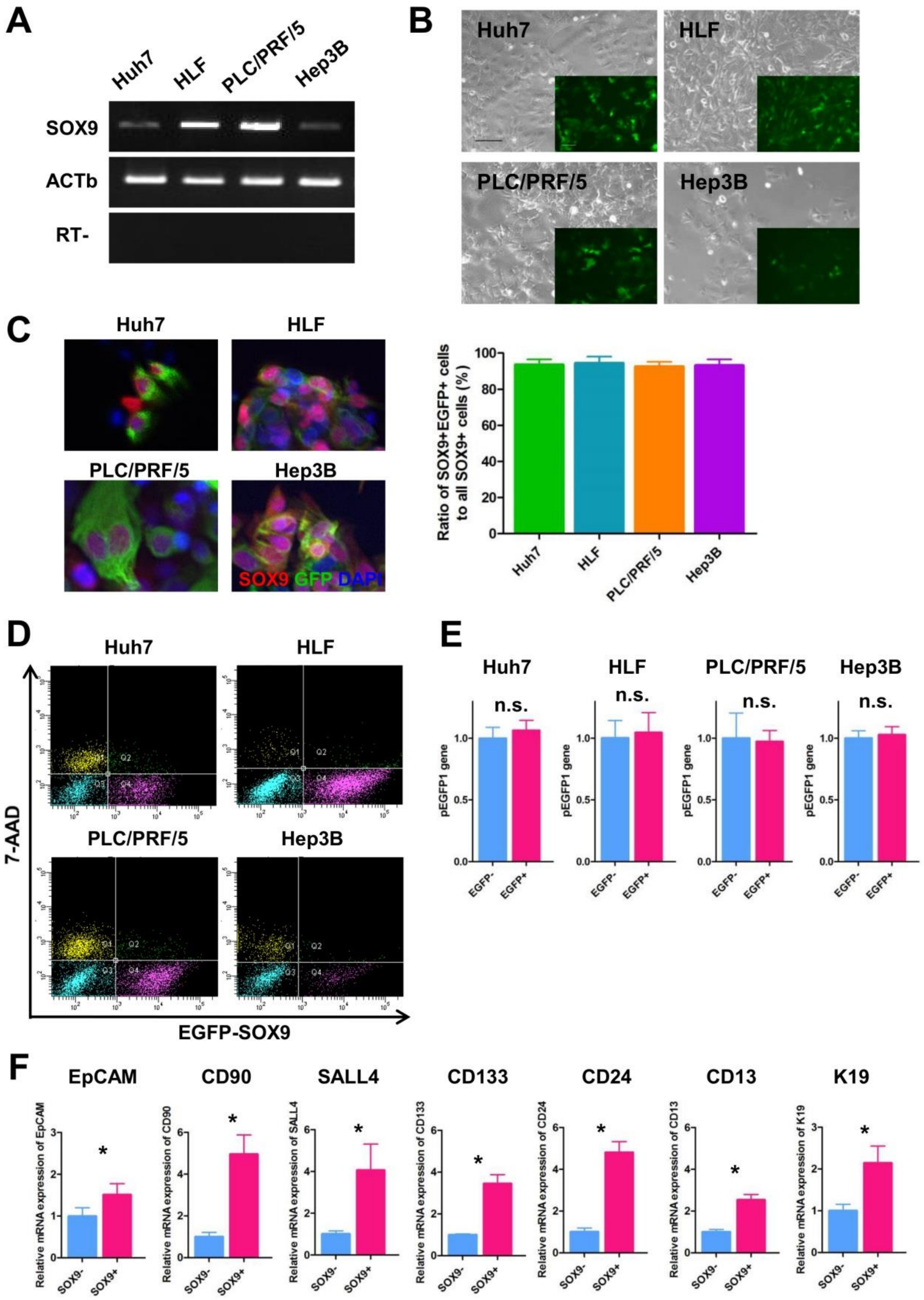

Supplemental Figure 2

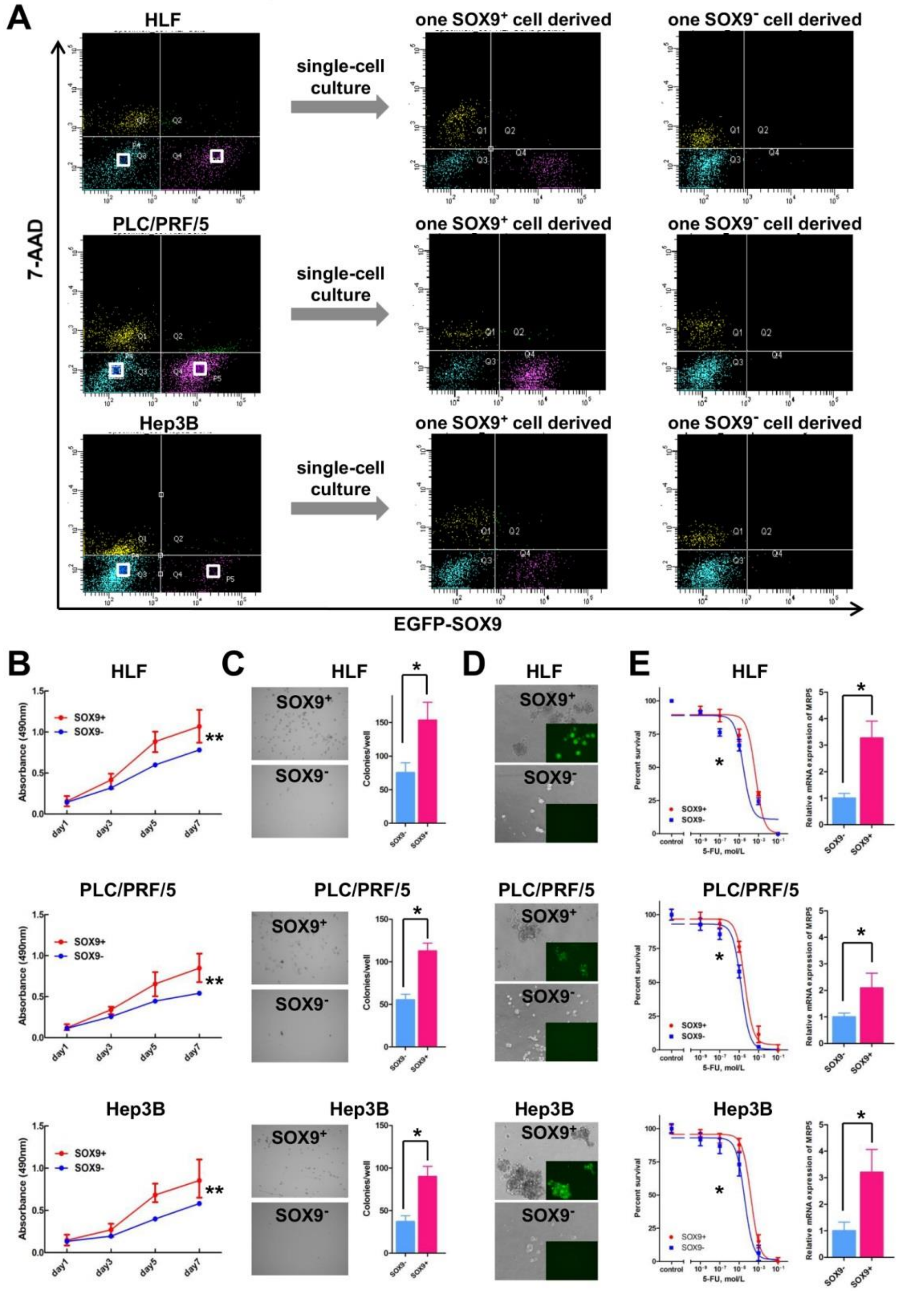

Supplemental Figure 3

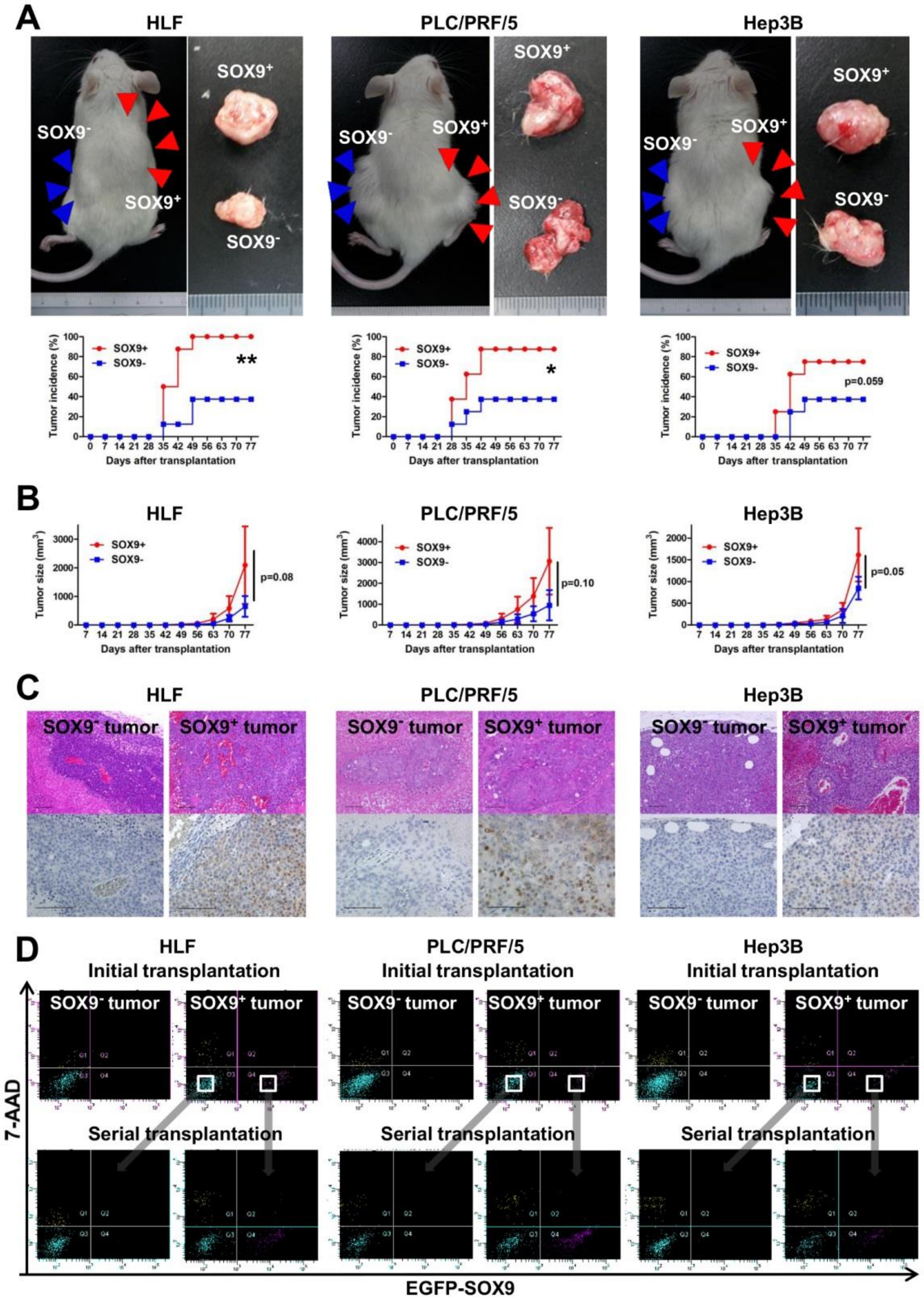

Supplemental Figure 4

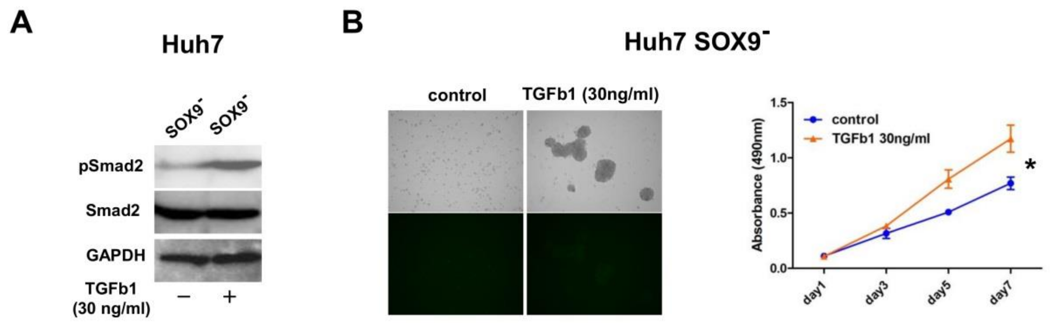

Supplemental Figure 5

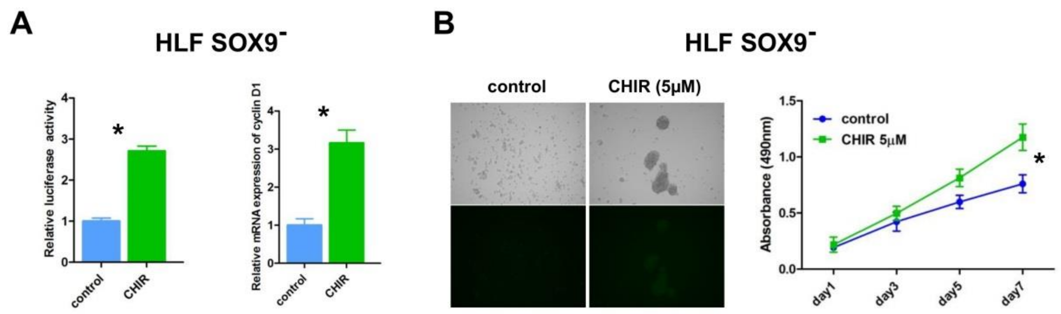

Supplemental Figure 6

A

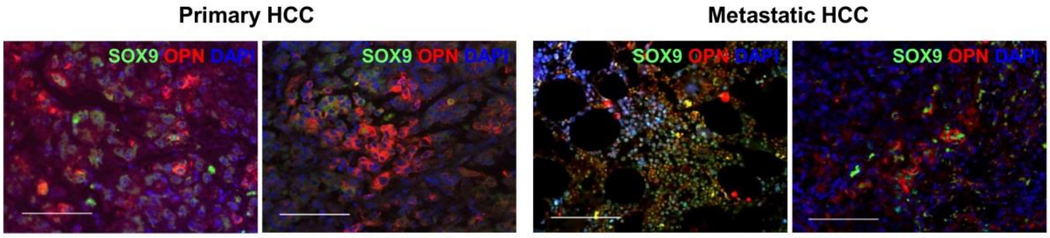

B

Primary HCC

Resection group

	OPN−	OPN+
SOX9−	52	15
SOX9+	6	31

** *P*<0.01

Transplantation group

	OPN−	OPN+
SOX9−	35	7
SOX9+	4	16

** *P*<0.01

Metastatic HCC

	OPN−	OPN+
SOX9−	2	0
SOX9+	0	9

** *P*<0.01

Supplemental Figure 7

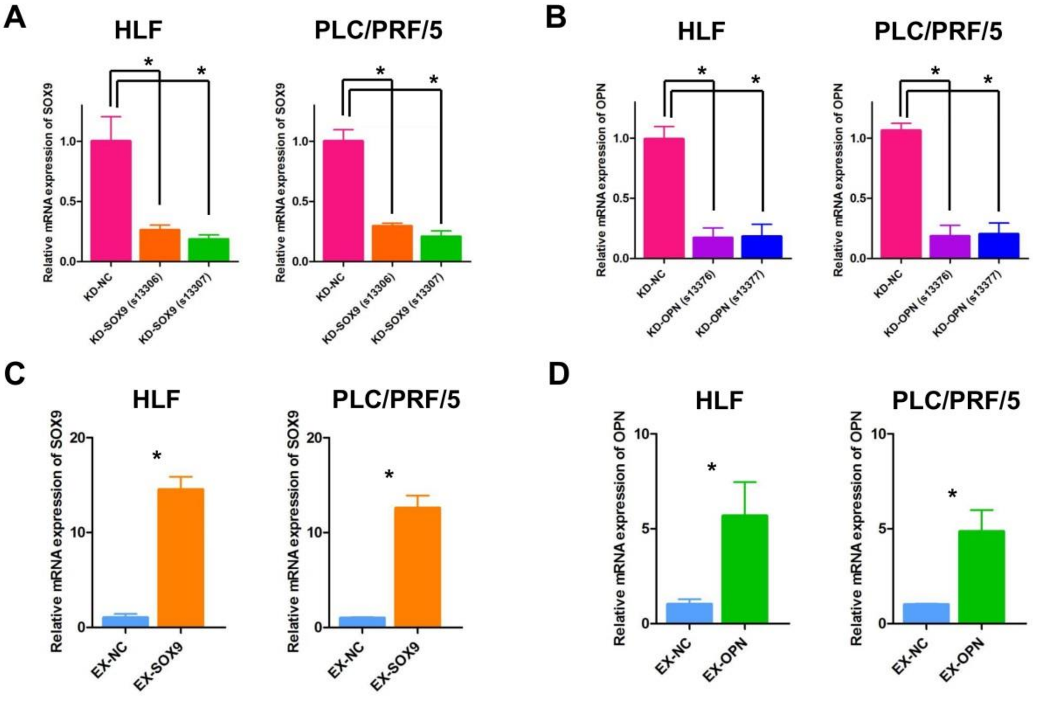

Supplement: Supplementary Information [file srep30489-s1.pdf]
